# Folate and global health review series, part 3: syntheses on cardiovascular, cerebrovascular, and metabolic diseases

**DOI:** 10.7189/jogh.16.04160

**Published:** 2026-05-11

**Authors:** Samantha Yoo, Azita Montazeri, Derrick Bennett, Yacong Bo, Peizhan Chen, Susan Duthie, Natalie Jensen, Atipatsa Kaminga, Jun-Shi Lai, Xue Li, Amanda J MacFarlane, Homero Martinez, Helene McNulty, Franco Momoli, Ron Munger, Rajendra Prasad Parajuli, Monique Potvin Kent, Michele Rubini, Marjanne Senekal, Lindsey Sikora, Alain Stintzi, Evropi Theodoratou, Hui Wang, Ann Yaktine, Julian Little

**Affiliations:** 1 School of Epidemiology and Public Health, Faculty of Medicine, University of Ottawa, Canada; 2 Nuffield Department of Population Health, University of Oxford, UK; 3 School of Public Health, Xinxiang Medical University, China; 4 Clinical Research Centre, Ruijin Hospital, Shanghai Jiao Tong University School of Medicine, Shanghai, China; 5 School of Pharmacy and Life Sciences, Robert Gordon University, Scotland, UK; 6 Department of Mathematics and Statistics, Faculty of Science, Technology and Innovation, Mzuzu University, Malawi; 7 Singapore Institute for Clinical Sciences, Agency for Science, Technology and Research, Singapore; 8 Centre for Population Health Sciences, University of Edinburgh, UK; 9 Nutrition Research Division, Health Canada, Canada; 10 Research and Development Unit, Nutrition International, Canada; 11 Nutrition Innovation Centre for Food and Health, School of Biomedical Sciences, Ulster University, Coleraine, Northern Ireland, UK; 12 School of Dentistry, University of Dundee, Scotland, UK; 13 Department of Nutrition, Dietetics, and Food Sciences, College of Agriculture and Applied Sciences, Utah State University, USA; 14 Herbert Wertheim School of Public Health and Human Longevity Science, University of California San Diego, USA; 15 Department of Neuroscience and Rehabilitation, University of Ferrara, Italy; 16 Department of Human Biology, Faculty of Health Sciences, University of Cape Town, South Africa; 17 Health Sciences Library, University of Ottawa, Canada; 18 School of Pharmacy, Faculty of Medicine, University of Ottawa, Canada; 19 Centre for Global Health, Usher Institute, College of Medicine and Veterinary Medicine, University of Edinburgh, UK; 20 School of Public Health, Faculty of Medicine, Shanghai Jiao Tong University, China; 21 Food and Nutrition Board, Health and Medicine Division, National Academies of Sciences, Engineering, and Medicine, USA

## Abstract

**Background:**

Cardiovascular and metabolic diseases account for an increasing share of morbidity and mortality globally. Folic acid supplementation has been linked to a lowered risk of stroke and some metabolic indicators due to its involvement in homocysteine and one-carbon metabolism and its role in the production of nitric oxide; however, the evidence on these associations is inconclusive.

**Methods:**

We searched MEDLINE, Embase, CINAHL, the Cochrane Library, and the Database of Abstracts of Reviews of Effects from inception to February 2024 for systematic reviews and meta-analyses investigating the associations of folate (dietary intake, supplementation, or blood concentrations) with any cardiometabolic outcome. We performed screening, data abstraction, and risk of bias assessment in duplicate, and assessed the credibility of the evidence using predefined criteria.

**Results:**

We identified 113 unique associations from 49 reviews. The included syntheses mostly had low risk of bias of and provided pooled risk estimates from intervention trials or prospective cohorts. A larger volume of evidence was available for composite cardiovascular outcomes, coronary heart disease, and stroke compared to other outcomes. No association reached a convincing or highly suggestive level of credibility. Six directional associations and five null associations met the criteria for a suggestive level of credibility. Three dose-response relationships, all at suggestive levels of credibility, supported an association between higher dietary folate intake and a reduced risk of coronary heart disease and stroke.

**Conclusion:**

The available evidence on the association between folate status and cardiometabolic outcomes primarily focuses on secondary prevention of cardiometabolic diseases and substantially underrepresents low- and middle-income countries. More large-scale studies are warranted to validate a relationship between folate status and cardiometabolic events or indicators. Overall, the evidence landscape around folate and cardiometabolic diseases appears to be limited both in volume and scope.

**Registration:**

PROSPERO: CRD42021265041.

Cardiovascular diseases (CVDs) encompass multiple subcategories of conditions, including coronary artery disease (CAD), heart failure, cerebrovascular diseases, cardiomyopathy, and peripheral artery disease. They are collectively the most prevalent chronic diseases globally as of 2019, accounting for approximately 523 million prevalent cases and 17.9 million deaths globally each year [1,2].

Metabolic diseases, an array of disorders including type 2 diabetes (T2D) and obesity, and risk factors such as hypertension and hyperlipidaemia have also been increasing globally [3]. There were approximately 43.8 million prevalent cases of T2D and 18.5 million cases of hypertension in 2019, together contributing to 2.5 million deaths [3,4]. High systolic blood pressure (SBP) and high low-density lipoprotein (LDL) cholesterol presented the highest risks for age-standardised all-cause mortality, followed by high fasting plasma glucose and high body mass index (BMI) [4]. Metabolic disorders often occur concurrently and are risk factors associated with CVDs [5–7], with associations between metabolic syndrome and cardiovascular morbidity and mortality being well-documented [8–11].

Folate has been studied in the context of treatment and management of many CVDs [12–15] due to its role in homocysteine and one-carbon metabolism. More specifically, the blood concentration of homocysteine is a well-established risk factor for vascular damage and atherosclerosis, and can be lowered with higher intakes of folate [16–20]. However, findings from randomised controlled trials (RCTs) of folic acid (FA) supplementation have been inconsistent, likely due to varying baseline levels of homocysteine, other potential confounders such as renal insufficiency, and background levels of FA fortification or supplementation [21,22]. The reported effect of FA supplementation on improving the status of metabolic markers is also inconclusive; some trials demonstrated a beneficial effect on insulin resistance and glycaemic control [23,24], while others showed no benefit [25,26].

In this umbrella review, we sought to critically synthesise the available evidence on relationships between folate status and risk of vascular and metabolic diseases across different populations.

## METHODS

We detailed the methodological framework used in this umbrella review in the first publication in this series [27]. In short, we searched MEDLINE, Embase, CINAHL, the Cochrane Library, and the Database of Abstracts of Reviews of Effects from inception to February 2024 for systematic reviews and meta-analyses that investigated associations between folate intake or status and any cardiovascular, cerebrovascular, or metabolic outcome. We screened the articles in two stages (title/abstract and full-text) and extracted data by two independent reviewers (SY, AM, NJ). All discrepancies were resolved by consensus. All of the included syntheses were assessed for methodological quality using the ROBIS tool (AM, NJ) [28].

We categorised the syntheses by type of exposure measure, outcome, and setting and identified unique associations (unique exposure– unique outcome– unique setting). Unique setting captured differences in the participant characteristics (baseline folate or homocysteine status, underlying comorbidities), FA fortification context, and primary *vs*. secondary prevention context. For each category of unique associations, we reviewed the syntheses for consistency in direction, magnitude, and statistical significance of the summary effects. If concordant, we selected the synthesis with the largest sample size. If discordant, we selected syntheses based on the largest total sample size, the largest number of cases (for binary outcomes), the recency of publication, and the highest methodological quality as assessed by ROBIS [28]. We evaluated the credibility of the selected evidence using predefined criteria (**Table 1**, **Table 2**).

**Table 1:** Criteria for credibility assessment

Category	Associations
**Directional associations**	
Convincing	
	With statistical significance of *P* < 10^−6^
	Based on ˃1000 cases (or ˃20 000 participants for continuous outcomes)
	For which largest component study reports a statistically significant result (*P* < 0.05) and has a 95% prediction interval that excludes the null
	Which do not have large heterogeneity (*I*^2^<50%)
	Show no evidence of small study effects (*P* ˃ 0.10) or of excess significance bias (*P* ˃ 0.10)
Highly suggestive	With statistical significance of *P* < 10^−6^
	Based on ˃1000 cases (or ˃20 000 participants for continuous outcomes)
	For which largest component study reports a statistically significant result (*P* < 0.05)
Suggestive	With statistical significance of *P* < 0.01
	Based on ˃1000 cases (or ˃20 000 participants for continuous outcomes)
Weak	With statistical significance of *P* < 0.05
**Null associations**	
Suggestive	Based on ˃1000 cases (or ˃20 000 participants for continuous outcomes)
	Which do not have large heterogeneity (*I*^2^<50%)
	With statistical significance of *P* > 0.10
Weak	With statistical significance of 0.05 < *P* < 0.10

**Table 2:** Worked examples of credibility assessment

Example 1	Li *et al*. [29] FA supplement – stroke, general population not otherwise stated		
	RR = 0.79 (0.69, 0.92), *P =* 0.001	Highly suggestive (*P* < 10^−6^)	N
		Suggestive (*P* < 0.01)	Y
	773 cases	Suggestive (>1000 cases)	N
	Credibility: Weak (does not meet the criteria for suggestive)		
**Example 2**	**Wang *et al*. [** 30 **] FA supplement – CHD, individuals with CVD**		
	RR = 1.08 (95% CI = 0.92, 1.27), *P =* 0.35	Suggestive (*P* > 0.10)	Y
	*I*^2^ = 0%	Suggestive (*I*^2^ < 50%)	Y
	Number of cases not reported	Suggestive (>1000 cases)	N
	Credibility: weak (downgraded from potentially suggestive due to insufficient data)		

CHD – chronic heart disease, CI – confidence interval, CVD – cardiovascular disease, N – no, RR – risk ratio, Y – yes

## RESULTS

### Overview of search results

A total of 49 out of 287 systematic reviews included in this umbrella review series [27] examined relationships between folate intake/status and risk of vascular and metabolic diseases, of which 46 provided meta-analyses. Forty meta-analyses consisted entirely of RCTs [12,29–67], four consisted of pooled prospective cohort studies [31,32,68,69], and two of case-control studies [70,71].

We examined five broad categories of outcomes across 113 unique associations in the meta-analyses (Tables S1 and S2 in the **Online Supplementary Document**): clinical events (cardiovascular events, coronary heart disease (CHD), stroke, myocardial infarction, thrombosis, mortality); vascular function surrogates (intima media thickness, flow mediated dilation, end-diastolic diameter, hyperaemic flow, arterial stiffness); and metabolic risk factors (blood glucose, fasting insulin, insulin resistance, blood pressure, cholesterol, body weight, BMI, polycystic ovary syndrome (PCOS)). We found larger volumes of evidence investigating CVDs (12 syntheses, 10 associations), CHD (8 syntheses, 9 associations), stroke (14 syntheses, 6 associations), elevated blood glucose levels (5 syntheses, 13 associations), elevated insulin levels (4 syntheses, 19 associations), and elevated blood pressure (5 syntheses, 10 associations) compared to other outcomes. We identified no meta-analysis for coronary restenosis.

FA supplementation was the most predominantly studied intervention (102 associations, 90%) across all outcome categories. Four of the associations were related to dietary folate intake (4%), two to total intake (2%), and five to plasma concentration (4%). In four associations, the authors aggregated dietary folate and total intakes.

### Risk of bias assessment

**Figure 1:**
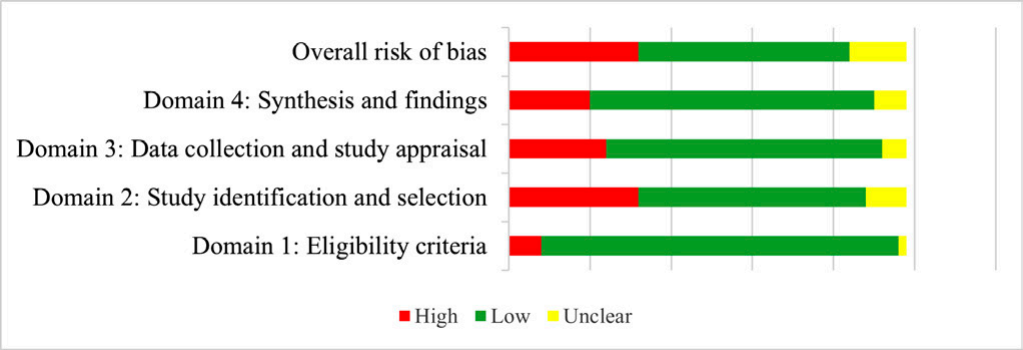
Risk of bias assessment of the included syntheses examining the relationship between folate intake/status and vascular/metabolic outcomes.

The risk of bias in the 49 included syntheses was generally low across the four domains, with low risk of bias in 26 (53.1%), high risk of bias in 16 (32.7%), and uncertain level of bias in 7 (14.3%) syntheses (**Figure 1**; Table S3 in the **Online Supplementary Document**). At the domain level, most of the syntheses were assessed as having low risk of bias in terms of the definitions of scope and eligibility criteria (89.8%), the identification and selection of studies (57.1%), data collection and appraisal (69.4%), and data synthesis (71.4%).

### Composite cardiovascular outcomes

Twelve meta-analyses [33–40,54,56,61,72] examined the effect of folic acid supplementation on cardiovascular outcomes in generally healthy populations or in individuals with underlying morbidities or risk factors. In the general population, FA supplementation significantly reduced the risk of CVDs (1627 cases; risk ratio (RR = 0.90; 95% confidence interval (CI) = 0.82, 0.99; *I*^2^ = 30%) compared to placebo or usual care [72]. Similarly, in a generally healthy population, FA (0.4–5 mg/d for 12–54 months) significantly reduced the risk of CVD (RR = 0.81; 95% CI = 0.70, 0.94; *I*^2^ = 0%) [33]. Among individuals with existing CVD or end stage renal disease (ESRD), supplementing with FA (0.5 mg/d to 15 mg/d for 6 months to 5 years) did not result in a change of CVD risk (14 440 participants, 2706 cases; RR = 0.95; 95% CI = 0.88, 1.03; *P*-value for heterogeneity <0.33) [34]. In individuals with a chronic kidney disease, the CVD risk was not different between the FA treatment group (5–15 mg/d for 12–43 months) and the placebo group (1180 participants, 348 cases; RR = 0.88; 95% CI = 0.75, 1.03; *I*^2^ = 0%) [35]. In individuals with CVD, ESRD, or a history of colorectal adenomas, FA supplementation (0.8–15 mg/d for 12–54 months) significantly reduced the risk of CVDs (21 567 participants, 960 cases; RR = 0.83; 95% CI = 0.73, 0.93; *I*^2^ = 0%) [36]. FA (5–15 mg/d for 12–29 months) was also more effective in reducing CVD risk in individuals undergoing dialysis (381 participants, 138 cases; RR = 0.73; 95% CI = 0.56, 0.94) [37]. Among Chinese individuals with hypertension or hyperhomocysteinaemia, but without history of stroke or myocardial infarction, adding FA supplementation (0.4–10 mg/d for 8 weeks to 18 months) significantly reduced the CVD risk (2057 participants, 300 cases; RR = 0.30; 95% CI = 0.21, 0.43; *I*^2^ = 43%) [38] compared to anti-hypertensive treatment alone.

Major adverse cardiovascular events (MACE) were examined separately in the general population, in individuals with CVD or ESRD, and in individuals with CHD, ESRD, or colorectal adenomas. The risk of MACE was not different between the FA group and the placebo group in the general population (0.5–15 mg/d for 12–43 months; 2871 participants, 282 cases; RR = 1.02; 95% CI = 0.84, 1.23; *I*^2^ = 47%) [39], in individuals with CVD or ESRD (0.5–5 mg/d for 24–87.6 months; 38 015 participants, 8238 cases; RR = 0.98; 95% CI = 0.93, 1.04; *I*^2^ = 31.8%) [40], and in individuals with CVD, ESRD, or colorectal adenomas (0.5–25 mg/d for 12–84 months; 4331 participants; RR = 1.02; 95% CI = 0.96, 1.08; *P*-value for heterogeneity = 0.48) [29].

### CHD

A higher intake of dietary folate was associated with a reduced risk of CHD in the general population (223 691 participants, 2682 cases; RR = 0.69; 95% CI = 0.60, 0.80; *I*^2^ = 0%) [32] and in individuals without history of CVD (232 549 participants, 3952 cases; RR = 0.68; 95% CI = 0.53, 0.84; *I*^2^ = 63%) [69].

The risk of CHD was not different between the FA supplementation and the placebo groups in the general population (RR = 1.13; 95% CI = 0.98, 1.31; *I*^2^ = 0%) [72], in individuals with CVD (0.5–5 mg/d for 12–60 months; 24 393 participants; RR = 1.08; 95% CI = 0.92, 1.27; *I*^2^ = 0%) [41], in individuals with CVD or ESRD (0.5–5 mg/d for 8.3–72 months; 19 050 participants, 3148 cases; RR = 1.06; 95% CI = 0.97, 1.15; *I*^2^ = 10.4%) [40], or in individuals with CVD, ESRD, or a history of colorectal adenomas (5–15 mg/d for 20–43 months; 2197 participants, 77 cases; RR = 1.47; 95% CI = 0.95, 2.28; *I*^2^ = 0%) [36].

A higher intake of total folate was associated with a reduced risk of CHD in the general population (308 012 participants; RR = 0.68; 95% CI = 0.57, 0.79) [31] and in individuals without a history of CVD (RR = 0.73; 95% CI = 0.59, 0.87; *I*^2^ = 0%) [69]. Plasma concentration of folate was not associated with CHD risk in the general population (14 533 participants, 1936 cases; RR = 0.74; 95% CI = 0.53, 1.02; *I*^2^ = 64.9%) [32].

### Stroke

A high intake of dietary folate was associated with a reduced risk of stroke (RR = 0.85; 95% CI = 0.78, 0.94) in a meta-analysis of prospective cohort studies of individuals with hypertension, T2D, or a history of stroke (255 458 participants, 7895 cases; *I*^2^ = 11.5%) [68].

Supplementation with FA was effective in reducing the risk of stroke in the general population (RR = 0.73; 95% CI = 0.69, 0.92; *I*^2^ = 11%) [72] and in individuals with CVD, ESRD, or a history of colorectal adenomas (0.5–5 mg/d for 12–120 months; 24 525 participants, 707 cases; RR = 0.79; 95% CI = 0.69, 0.92; *I*^2^ = 0%) [36]. In individuals with CVD, ESRD, or oesophageal dysplasia mostly living in countries without mandatory FA fortification, the risk of stroke was lower in the FA supplementation group compared to the placebo group (28 450 participants, 827 cases; RR = 0.75; 95% CI = 0.66, 0.86; *I*^2^ = 0%) [42]. In individuals with CVD or ESRD, FA supplementation did not reduce the risk of stroke (0.5–5 mg/d for 12–72 months; 42 960 participants, 2001 cases; RR = 0.89; 95% CI = 0.78, 1.01; *I*^2^ = 31.6%) [40]. This finding was consistent in stratified analyses where the association was examined by the FA fortification status of the countries studied, *i.e.* in countries with mandatory fortification (2.5–40 mg/d for 32–87 months; 8051 participants, 258 cases; RR = 0.94; 95% CI = 0.58, 1.54; *I*^2^ = 61%), in countries without mandatory fortification (0.2–2 mg/d for 24–78 months; 24 260 participants, 846 cases; RR = 0.91; 95% CI = 0.82, 1.01; *I*^2^ = 1%), and in studies conducted across countries with or without mandatory fortification (0.02–2.5 mg/d for 24–60 months; 17 366 participants, 1306 cases; RR = 0.88; 95% CI = 0.77, 1.00; *I*^2^ = 47%) [12].

### Thrombosis

One meta-analysis of case-control studies [70] examined relationships between plasma concentration of folate and four thrombosis outcomes. A higher plasma concentration of folate was associated with reduced risk of venous thrombosis (4197 participants, 1937 cases; standardised mean difference (SMD) = −0.55; 95% CI = −0.75, −0.36; *I*^2^ = 88%), deep vein thrombosis/pulmonary thromboembolism (2632 participants, 1250 cases; SMD =  −0.42; 95% CI = −0.66, −0.17; *I*^2^ = 87%), cerebral venous thrombosis (622 participants, 226 cases; SMD = −0.61; 95% CI = −0.98, −0.25; *I*^2^ = 75%), and retinal vein occlusion (752 participants, 398 cases; SMD = −0.39; 95% CI = −0.60, −0.19; *I*^2^ = 31%).

### Myocardial infarction

FA supplementation did not show an effect in reducing the risk of myocardial infarction in individuals with CVD or ESRD (0.5–5 mg/d for 8.3–72 months; 39 923 participants, 2917 cases; RR = 1.00; 95% CI = 0.93, 1.07; *I*^2^ = 0%) [40] or among individuals with CVD, ESRD, or history of colorectal adenomas (0.5–5 mg/d for 12–64.1 months; 24 210 participants, 140 cases; RR = 1.24; 95% CI = 0.87, 1.25; *I*^2^ = 4%) [36].

### Revascularisation

The risk of revascularisation in individuals with CVD or ESRD was not different between the placebo and the FA supplementation groups (0.5–5 mg/d for 8.3–60 months; 38 068 participants; RR = 1.05; 95% CI = 0.95, 1.16; *I*^2^ = 40%) [40].

### Coronary restenosis

Three RCTs of FA interventions on individuals with stable CAD or who underwent angioplasty procedures were synthesised narratively [73]. The effect of FA on the rate of coronary restenosis was inconclusive.

### Vascular mortality

Zhou *et al*. [40] reported that supplementing FA in individuals with CVD or ESRD reduced the risk of vascular death (27 342 participants; RR = 0.89; 95% CI = 0.81, 0.98; *I*^2^ = 0%). However, in individuals with CVD, ESRD, or a history of colorectal adenomas, there was no clear link between FA supplementation and cardiovascular death (0.5–15 mg/d for 24–54 months; 22 468 participants, 201 cases; RR = 0.87; 95% CI = 0.67, 1.13; *I*^2^ = 0%), myocardial infarction death (0.8–5 mg/d for 12–54 months; 20 985 participants, 13 cases; RR = 1.17; 95% CI = 0.38, 3.49; *I*^2^ = 0%), and stroke death (0.8–5 mg/d for 12–54 months; 20 985 participants, 29 cases; RR = 1.85; 95% CI = 0.88, 3.93; *I*^2^ = 0%) [36].

### All-cause mortality

Four meta-analyses [33,34,36,40] investigated the effect of FA supplementation on reducing mortality. Total mortality was not different between FA intervention and placebo groups in a generally healthy population (0.8–5 mg/d for 37–54 months; RR = 1.00; 95% CI = 0.60, 1.99; *I*^2^ = 37%) [33], among individuals with CVD or ESRD (0.5–5 mg/d for 8.3–72 months; 44 340 participants, 6314 cases; RR = 1.00; 95% CI = 0.96, 1.05; *I*^2^ = 0%) [39], or among individuals with CVD, ESRD, or history of colorectal adenomas (0.5–15 mg/d for 12–120 months; 25 580 participants, 895 cases; RR = 0.84; 95% CI = 0.69, 1.03; *I*^2^ = 23%) [36].

### Endothelial function

Six reviews [43,45,46,55,63,66] reported on the effect of FA supplementation on different measures of endothelial function in individuals with CVD or risk factors for CVD. There was no evidence of an effect of FA intervention (5 mg/d for 4–16 weeks) compared to placebo on composite measure of endothelial function in individuals with CAD (1796 participants; MD = 0.00; 95% CI = −0.17, 0.18; *I*^2^ = 88%) [43]. Compared to placebo, FA treatment was effective in reducing the carotid intima-media thickness (0.8–15 mg/d for 3–42 months; 1459 participants; WMD = −0.04 mm; 95% CI = −0.06, −0.01) in individuals with chronic kidney disease or CVD risks [44], improving the percent flow-mediated dilation change (%FMD) (0.4–10 mg/d for 4–52 months; 712 participants; %FMD = 1.11%; 95% CI = 0.60, 1.62) [45], but not in improving FMD in individuals with or without CVD or ESRD (0.4–10 mg/d for 2–17 weeks; 601 participants; SMD = 0.89; 95% CI = 0.45, 1.33; *I*^2^ = 82.9%) [46]. No difference was reported between the FA (5 mg/d for 4–16 weeks) and placebo groups in terms of end diastolic diameter (237 participants; MD = −0.03; 95% CI = −0.20, 0.15; *I*^2^ = 0%), glyceryl trinitrate-mediated dilation (187 participants; MD = 1.74 *µ*m; 95% CI = −17.13, 20.61; *I*^2^ = 0%), baseline hypaeremic flow (187 participants; MD = 1.02 ml/min; 95% CI = −4.81, 6.84; *I*^2^ = 0%), or peak hypaeremic flow (187 participants; MD = −2.25 ml/min; 95% CI = −23.31, 18.82; *I*^2^ = 0%) [43].

### Arterial stiffness

Pulse wave velocity (PWV) was not different between the FA treatment and placebo groups of individuals in the UK with or without T2D (5 mg/d for 4 weeks; 50 participants; MD = −0.14 m/s; 95% CI = −0.69, 0.42) [47]. Central PWV (SMD = −0.07; 95% CI = −0.26, 0.13; *I*^2^ = 0%) or peripheral PWV (SMD = −0.09; 95% CI = −0.26, 0.08; *I*^2^ = 15.2%) measurements also did not change with FA supplementation (5–15 mg/d for 4–206 weeks), compared to placebo, in individuals with cardiometabolic conditions or chronic kidney disease [46].

### Blood pressure

Five meta-analyses [38,43,48,52,63] examined the effect of FA supplementation in inducing changes in blood pressure. In individuals with either cardiometabolic disorders or risk factors, FA treatment significantly lowered both SBP (41 633 participants; WMD = −1.11 mmHg; 95% CI = −1.93, −0.28; *I*^2^ = 65%) and diastolic blood pressure (DBP) (41 589 participants; WMD = −0.24 mmHg; 95% CI = −0.37, −0.11; *I*^2^ = 47.9%) compared to the placebo group [48]. The effect of FA in lowering SBP was observed among subgroups of individuals with a baseline SBP ≥120 mmHg (WMD = −1.16 mmHg; 95% CI = −2.05, −0.27), with pre-existing CVD (WMD = −1.08 mmHg; 95% CI = −2.10, −0.06), with a BMI of 25.0–29.9 (WMD = −2.43 mmHg; 95% CI = −4.22, −0.63), and in both males (WMD = −3.10 mmHg; 95% CI = −6.09, −0.11) and females (WMD = −2.81 mmHg; 95% CI = −5.45, −0.16). The effect of FA supplementation on reducing DBP was observed in individuals with pre-existing CVD (WMD = −0.23 mmHg; 95% CI = −0.36, −0.09), with a BMI of 18.5–24.9 (WMD = −0.30 mmHg; 95% CI = −0.47, −0.13), and in males (WMD = −4.19 mmHg; 95% CI = −7.51, −0.86).

In 5707 Chinese individuals with a diagnosis of hypertension and hyperhomocysteinaemia, but without a history of stroke or myocardial infarction, the use of FA supplements (0.4–10 mg/d for 8 weeks to 18 months) in addition to antihypertensive medications was reported to reduce SBP (WMD = −7.85; 95% CI = −9.43, −6.27; *I*^2^ = 95%) and DBP (WMD = −6.77; 95% CI = −8.55, −5.00; *I*^2^ = 97%) compared to using antihypertensives only [38].

### Heart rate

One meta-analysis reported that FA supplementation (5 mg/d for 4–16 weeks), compared to placebo, had no effect on heart rate (237 participants; MD = −0.39 beats/min; 95% CI = −2.89, 2.11; *I*^2^ = 0%) [43].

### Blood glucose

FA supplementation did not have a clear effect on improving haemoglobin A1c (HbA1c) in individuals with cardiometabolic conditions or cervical intraepithelial neoplasia (0.15–5 mg/d for 4 weeks to 6 months; 313 participants; MD = −0.17%; 95% CI = −0.49, 0.16; *I*^2^ = 77.8%) [30] or in Iranian individuals with T2D (5 mg/d for 4–8 weeks; 142 participants; WMD = −0.37 *µ*mol/L; 95% CI = −1.10, 0.35; *I*^2^ = 83.8%) [49]. Supplementing FA (0.25–10 mg/d for 2–234 weeks) was beneficial in lowering the fasting glucose in individuals with CVD, T2D, PCOS, endometrial hyperplasia or menopause (34 593 participants; WMD = −2.17; 95% CI = −3.69, −0.65; *I*^2^ = 81.5%) [50]. This benefit was observed in the subgroup analyses of individuals with the baseline fasting glucose <100 mg/dL (12 547 participants; WMD = −2.14; 95% CI = −4.36, −0.06; *I*^2^ = 81.5%), individuals with the baseline fasting glucose ≥100 mg/dL (22 067 participants; WMD = −4.06; 95% CI = −7.83, −0.29); *I*^2^ = 71.9%), individuals without T2D (21 335 participants; WMD = −2.34; 95% CI = −4.46, −0.22; *I*^2^ = 83.7%), and both males (116 participants; WMD = −18.81; 95% CI = −26.87, −10.74; *I*^2^ = 0%) and females (455 participants; WMD = −9.53; 95% CI = 14.71, −4.35; *I*^2^ = 90.8%). FA was also reported to be effective in lowering the fasting glucose in studies with follow-ups of less than 12 weeks (1126 participants; WMD = −5.32; 95% CI = −9.11, −1.53; *I*^2^ = 86.5%) and in studies using a dose ≥5 mg/d (849 participants; WMD = −3.58; 95% CI = −6.62, −0.54); *I*^2^ = 78.3%) [50].

### Insulin

In individuals with cardiometabolic conditions, FA intervention lowered insulin (1–10 mg/d for 4–12 weeks; 453 participants; SMD = −1.28; 95% CI = −1.99, −0.56; *I*^2^ = 91.5%) [51] and fasting insulin levels (0.4–15 mg/d for 3–25 weeks; 633 participants; WMD = −1.63 *µ*U/mL; 95% CI = −2.53, −0.73; *I*^2^ = 74.9%) compared to placebo [50]. This benefit was observed in subgroups of individuals without T2D (490 participants; WMD = −1.96 *µ*U/mL; 95% CI = −2.92, −1.00; *I*^2^ = 65.3%) and females (370 participants; WMD = −2.01 *µ*U/mL; 95% CI = −3.14, −0.88; *I*^2^ = 69%). Benefit was also seen in studies with a follow-up duration ≥12 weeks (359 participants; WMD = −2.03 *µ*U/mL; 95% CI = −3.31, −0.75; *I*^2^ = 55.8%), and in studies using FA doses <5 mg/d (148 participants; WMD = −0.99 *µ*U/mL; 95% CI = −1.94, −0.04; *I*^2^ = 0.9%) or ≥5 mg/d (458 participants; WMD = −1.86*µ*U/mL; 95% CI = −3.00, −0.71; *I*^2^ = 70.5%) [50]. FA supplementation (0.3–15 mg/d for 3–25 weeks) was reported to lower insulin resistance (HOMA-IR), compared to placebo in individuals with cardiometabolic conditions (644 participants; WMD = −0.40 units; 95% CI = −0.70, −0.09; *I*^2^ = 80.9%) [50]. This benefit was consistently observed in subgroups of individuals without T2D (501 participants; WMD = −0.43 units; 95% CI = −0.77, −0.08; *I*^2^ = 83.9%); and in studies with a follow-up of less than 12 weeks (227 participants; WMD = −0.62 units; 95% CI = −0.64, −0.59; *I*^2^ = 0%) and with a dose ≥5 mg/d (438 participants; WMD = −0.62 units; 95% CI = −0.64, −0.60; *I*^2^ = 0%).

### Cholesterol

One meta-analysis examined the effect of FA intervention (2.5–10 mg/d for 2–12 weeks) on improving different types of cholesterol in individuals with cardiometabolic conditions [52] and found no difference between FA and placebo groups in measurements of triglycerides (542 participants; SMD = 0.10; 95% CI = −0.42, 0.63; *I*^2^ = 88%), total cholesterol (492 participants; SMD = 0.06; 95% CI = −0.31, 0.43; *I*^2^ = 74.9%), LDL cholesterol (432 participants; SMD = −0.14; 95% CI = −0.55, 0.28; *I*^2^ = 77.1%), high-density lipoprotein cholesterol (492 participants; SMD = 0.04; 95% CI = −0.36, 0.44; *I*^2^ = 78.7%), and very LDL cholesterol (145 participants; SMD = 0.08; 95% CI = −0.24, 0.41; *I*^2^ = 0%).

### Body weight and BMI

Among individuals with metabolic conditions, FA supplementation (1.5–15 mg/d for 3–24 weeks) did not have a conclusive effect on reducing body weight or BMI compared to placebo (378 participants; WMD = −0.16 kg; 95% CI = −0.47, 0.16; *I*^2^ = 40.4%) [53]. FA supplementation (1.5–7.5 mg/d for 3–12 weeks) was also not effective in lowering BMI in individuals with metabolic conditions, or menopause (436 participants; WMD = −0.23kg/cm^2^; 95% CI = −0.49, 0.03; *I*^2^ = 90.1%) or in a subgroup of individuals with homocysteine ≥15 *µ*mol/L (WMD = −0.17kg/cm^2^; 95% CI = −0.33, 0.01). However, a benefit was reported in a subgroup of women with PCOS (WMD = −0.30kg/cm^2^; 95% CI = −0.54, −0.06).

### PCOS

One meta-analysis [71] compared dietary folate intake in women with PCOS compared with healthy controls. The authors did not find significant difference between the two groups (9562 participants, 1161 cases; MD = −20.80 *µ*g/d; 95% CI = −42.65, 1.05; *I*^2^ = 37.5%).

### Dose-response relationship

Three dose-response relationships were reported for CHD and one for stroke. Pooling from seven prospective cohort studies of the general population (223 691 participants, 2682 cases), Wang *et al*. [32] reported that the risk of CHD was reduced for every 200 *µ*g/d increase in the intake of dietary folate (RR = 0.88; 95% CI = 0.82, 0.94; *I*^2^ = 27.4%). From eight prospective cohort studies (14 533 participants, 1936 cases), the same authors reported that the risk of CHD was reduced for every 5 mmol/L increase in the plasma concentration of folate (RR = 0.92; 95% CI = 0.84, 1.00; *I*^2^ = 51.7%). In individuals without history of CVD, the risk of CHD was reduced (RR = 0.79; 95% CI = 0.69, 0.89; *I*^2^ = 67%) for every 25 *µ*g/d increase in the intake of dietary or total folate based on seven prospective cohort studies (212 284 participants, 4022 cases) [69]. The risk of stroke was reduced for every 100 *µ*g/d increase in the intake of dietary folate (RR = 0.94; 95% CI = 0.90, 0.98; *I*^2^ = 46.8%) in individuals with CVD (eight prospective cohort studies, 253 511 participants, 7429 cases) [68].

### Credibility assessment

Of the 113 unique associations, six directional associations and five null associations were assessed to be of suggestive level of credibility. A total of 20 associations were downgraded to a lower level of credibility due to uncertainty in the statistical power; another 84 associations were of weak credibility; and no association reached a convincing or highly suggestive level. Small samples size or high heterogeneity were the predominant reasons for grading the evidence as weak (**Table 3**).

**Table 3:** Identified unique associations between folate intake/status and vascular/metabolic diseases

Author (year)	Outcome (setting/subgroup)	Primary study design	Exposure	Total number (number of cases)	Metric	Summary estimate (95% CI)	Estimated *P*-value	*I*^2^ (%)	Credibility
Schwingshackl *et al*. (2017) [33]	CVD, generally healthy population	RCT	Supplement	NR (NR)	RR	0.81 (0.70, 0.94)	0.00507879	0	Weak (insufficient data)
Li *et al*. (2016) [72]	CVD, general population not otherwise stated	RCT	Supplement	NR (1627)	RR	0.90 (0.82, 0.99)	0.02836452	30	Weak
Bazzano *et al*. (2006) [34]	CVD, individuals with history of CVD or ESRD	RCT	Supplement	14 440 (2706)	RR	0.95 (0.88, 1.03)	0.20142306	71.2	Suggestive
Pan *et al*. (2012) [35]	CVD, individuals with ESRD (with or without dialysis)	RCT	Supplement	1180 (348)	RR	0.88 (0.75, 1.03)	0.11420346	0	Weak
Heinz *et al*. (2009) [37]	CVD, individuals with ESRD undergoing dialysis	RCT	Supplement	381 (138)	RR	0.73 (0.56, 0.94)	0.01722561	NR	Weak
Jenkins *et al*. (2021) [36]	CVD, individuals with CVD, ESRD or history of colorectal adenomas	RCT	Supplement	21 567 (960)	RR	0.83 (0.73, 0.93)	0.00255723	0	Weak
Wang *et al*. (2017) [38]	CVD, Chinese individuals with hypertension and hyperhomocysteinaemia without history of stroke or MI	RCT	Supplement	2057 (300)	RR	0.30 (0.21, 0.43)	0.00000000	43	Weak
Myung *et al*. (2013) [39]	MACE, general population not otherwise stated	RCT	Supplement	2871 (282)	RR	1.02 (0.84, 1.23)	0.83870736	47	Weak
Zhou *et al*. (2011) [40]	MACE, individuals with CVD or ESRD	RCT	Supplement	38 015 (8238)	RR	1.00 (0.93, 1.04)	0.94403381	31.8	Suggestive
Zhang *et al*. (2014) [29]	MACE, individuals with CHD, ESRD, or colorectal adenomas	RCT	Supplement	4331 (NR)	RR	1.02 (0.96, 1.08)	0.50985609	55.9	Weak
Wang *et al*. (2012) [32]	CHD, general population not otherwise stated	PC	Dietary folate	223 691 (2682)	RR	0.69 (0.60, 0.80)	0.00000043	0	Suggestive
Jayedi (2019) [69]	CHD, individuals without history of CVD	PC	Dietary folate	232 549 (3952)	RR	0.68 (0.53, 0.84)	0.00102793	63	Suggestive
Li *et al*. (2016) [72]	CHD, general population not otherwise stated	RCT	Supplement	NR (689)	RR	1.13 (0.98, 1.31)	0.09879231	0	Weak
Wang *et al*. (2019) [41]	CHD, individuals with CVD	RCT	Supplement	24 393 (NR)	RR	1.08 (0.92, 1.27)	0.34939733	0	Weak (insufficient data)
Zhou *et al*. (2011) [40]	CHD, individuals with CVD or ESRD	RCT	Supplement	19 050 (3148)	RR	1.06 (0.97, 1.15)	0.17963917	10.4	Suggestive
Jenkins *et al*. (2021) [36]	CHD, individuals with CVD, ESRD or history of colorectal adenomas	RCT	Supplement	2197 (77)	RR	1.47 (0.95, 2.28)	0.08451820	0	Weak
Mente *et al*. (2009) [31]	CHD, general population not otherwise stated	PC	Total folate	308 012 (NR)	RR	0.68 (0.57, 0.79)	0.00000363	NR	Weak (insufficient data)
Jayedi *et al*. (2019) [69]	CHD, individuals without history of CVD	PC	Total folate	NR (NR)	RR	0.73 (0.59, 0.87)	0.00149055	0	Weak (insufficient data)
Wang *et al*. (2012) [32]	CHD, general population not otherwise stated		Plasma folate	14 533 (1936)	RR	0.74 (0.53, 1.02)	0.07140206	64.9	Weak
Chen *et al*. (2020) [68]	Stroke, individuals with hypertension, T2D, or history of stroke	PC	Dietary folate	255 458 (7895)	RR	0.85 (0.78, 0.94)	0.00063929	11.50	Suggestive
Li *et al*. (2016) [72]	Stroke, general population not otherwise stated	RCT	Supplement	NR (773)	RR	0.79 (0.69, 0.92)	0.00131820	11	Weak
Zhou *et al*. (2011) [40]	Stroke, individuals with CVD or ESRD	RCT	Supplement	42 960 (2001)	RR	0.89 (0.79, 1.01)	0.07709928	31.9	Weak
Hsu *et al*. (2018) [42]	Stroke, individuals with CVD, ESRD, or esophageal dysplasia mostly living in countries without FA fortification	RCT	Supplement	28 450 (827)	RR	0.75 (0.66, 0.86)	0.00002040	0	Weak
Zeng *et al*. (2015) [12]	Stroke, individuals with cardiometabolic or renal conditions living in countries with fortification	RCT	Supplement	8051 (258)	RR	0.94 (0.58, 1.54)	0.80383555	61	Weak
Zeng *et al*. (2015) [12]	Stroke, individuals with cardiometabolic or renal conditions living in countries with partial fortification	RCT	Supplement	17 366 (1306)	RR	0.91 (0.82, 1.01)	0.07606790	47	Weak
Zeng *et al*. (2015) [12]	Stroke, individuals with cardiometabolic or renal conditions living in countries without fortification	RCT	Supplement	24 260 (846)	RR	0.88 (0.77, 1.00)	0.05520364	1	Weak
Jenkins *et al*. (2021) [36]	Stroke, individuals with CVD, ESRD, or history of colorectal adenoma	RCT	Supplement	24 525 (707)	RR	0.79 (0.69, 0.92)	0.00131820	NR	Weak
Zhou *et al*. (2012) [70]	VT	CC	Plasma folate	4197 (1937)	SMD	−0.55 (−0.75, −0.37)	0.00000001	88	Weak
Zhou *et al*. (2012) [70]	DVT/PTE	CC	Plasma folate	2632 (1250)	SMD	−0.42 (−0.66, −0.17)	0.00077942	70.3	Weak
Zhou *et al*. (2012) [70]	CVT	CC	Plasma folate	622 (226)	SMD	−0.61 (−0.98, −0.25)	0.00105432	0	Weak
Zhou *et al*. (2012) [70]	RVO	CC	Plasma folate	752 (398)	SMD	−0.39 (−0.60, −0.19)	0.00019241	0	Weak
Zhou *et al*. (2011) [40]	MI, individuals with CVD or ESRD	RCT	Supplement	39 923 (2917)	RR	1.00 (0.93, 1.07)	1.00000000	0	Suggestive
Jenkins *et al*. (2021) [36]	MI, individuals with CVD or ESRD or history of colorectal adenoma	RCT	Supplement	24 210 (140)	RR	1.24 (0.87, 1.75)	0.22760244	4	Weak
Zhou *et al*. (2011) [40]	Revascularisation, individuals with CVD or ESRD	RCT	Supplement	38 068 (NR)	RR	1.05 (0.95, 1.16)	0.33823422	40	Weak (insufficient data)
Zhou *et al*. (2011) [40]	Vascular mortality, individuals with CVD or ESRD	RCT	Supplement	27 342 (NR)	RR	0.89 (0.81, 0.98)	0.01649677	0	Weak
Jenkins *et al*. (2021) [36]	CVD mortality, individuals with CVD, ESRD or history of colorectal adenomas	RCT	Supplement	22 468 (201)	RR	0.87 (0.7, 1.13)	0.29629644	0	Weak
Jenkins *et al*. (2021) [36]	MI mortality, individuals with CVD, ESRD or history of colorectal adenomas	RCT	Supplement	20 985 (13)	RR	1.17 (0.39, 3.49)	0.77883629	0	Weak
Jenkins *et al*. (2021) [36]	Stroke mortality, individuals with CVD, ESRD or history of colorectal adenomas	RCT	Supplement	20 985 (29)	RR	1.85 (0.88, 3.93)	0.10707635	0	Weak
Jenkins *et al*. (2021) [36]	Total mortality, generally healthy population	RCT	Supplement	NR (NR)	RR	1.00 (0.60, 1.99)	1.00000000	23.8	Weak (insufficient data)
Jenkins *et al*. (2021) [36]	Total mortality, individuals with CVD or ESRD	RCT	Supplement	44 340 (6314)	RR	1.00 (0.96, 1.05)	1.00000000	0	Suggestive
Jenkins *et al*. (2021) [36]	Total mortality, individuals with CVD, ESRD or history of colorectal adenomas	RCT	Supplement	25 580 (895)	RR	0.84 (0.69, 1.03)	0.08800654	23	Weak
Yi *et al*. (2014) [43]	Endothelial function, individuals with CAD	RCT	Supplement	1796 (NA)	MD	0.00 (−0.17, 0.18)	1.00000000	88	Weak
Qin *et al*. (2012) [44]	CIMT, individuals with CVD, CVD risk or CKD	RCT	Supplement	1459 (NA)	WMD	−0.04 (−0.06, −0.01)	0.00171269	NR	Weak
Debree *et al*. (2007) [45]	FMD, individuals with CVD or CVD risk	RCT	Supplement	712 (355)	Diff	1.11 (0.60, 1.62)	0.00001991	NR	Weak
Bokayeva *et al*. (2023) [46]	FMD, individuals with CVD, ESRD or healthy	RCT	Supplement	601 (NA)	SMD	0.89 (0.45, 1.33)	0.00007354	82.9	Weak
Yi *et al*. (2014) [43]	EDD, individuals with CAD	RCT	Supplement	237 (NA)	MD	−0.33 (−0.20, 0.15)	0.73687087	0	Weak
Yi *et al*. (2014) [43]	GTN, individuals with CAD	RCT	Supplement	187 (NA)	MD	1.74 (−17.13, 20.61)	0.85657847	0	Weak
Yi *et al*. (2014) [43]	Baseline hyperemic flow, individuals with CAD	RCT	Supplement	187 (NA)	MD	1.02 (−4.81, 6.84)	0.73144026	0	Weak
Yi *et al*. (2014) [43]	Peak hyperemic flow, individuals with CAD	RCT	Supplement	187 (NA)	MD	−2.25 (−23.31, 18.82)	0.83417346	0	Weak
Saz-Lara *et al*. (2022) [47]	PWV, individuals in UK with or without T2D	RCT	Supplement	50 (NA)	MD	−0.14 (−0.69, 0.42)	0.62101354	NR	Weak
Bokayeva *et al*. (2023) [46]	Central PWV, individuals with CVD, CVD risk or ESRD	RCT	Supplement	421 (NA)	SMD	−0.07 (−0.26, 0.13)	0.48168830	0	Weak
Bokayeva *et al*. (2023) [46]	Peripheral PWV, individuals with T2D or ESRD	RCT	Supplement	553 (NA)	SMD	−0.09 (−0.26, 0.08)	0.29943440	15.2	Weak
Asbaghi *et al*. (2023) [48]	SBP, individuals with CVD, T2D, PCOS, HIV, menopause or alcohol/cigarette use	RCT	Supplement	41 633 (20 896)	WMD	−1.11 (−1.93, −0.28)	0.00836204	65	Suggestive
	*Baseline <120 mmHg*	RCT	Supplement	NR (NA)	WMD	−1.03 (−3.05, 0.98)	0.31639872	NR	Weak (insufficient data)
	*Baseline ≥120 mmHg*	RCT	Supplement	NR (NA)	WMD	−1.16 (−2.05, −0.27)	0.01063078	NR	Weak (insufficient data)
	*Individuals with CVD*	RCT	Supplement	NR (NA)	WMD	−1.08 (−2.10, −0.06)	0.03795928	NR	Weak
	*Individuals without CVD*	RCT	Supplement	NR (NA)	WMD	−0.97 (−2.20, 0.25)	0.12066222	NR	Weak
	*Male*	RCT	Supplement	NR (NA)	WMD	−3.10 (−6.09, −0.11)	0.04214282	NR	Weak
	*Female*	RCT	Supplement	NR (NA)	WMD	−2.81 (−5.45, −0.16)	0.03731796	NR	Weak
	*BMI 18.5–24.9*	RCT	Supplement	NR (NA)	WMD	−0.53 (−1.77, 0.69)	0.39836055	NR	Weak (insufficient data)
	*BMI 25–29.9*	RCT	Supplement	NR (NA)	WMD	−2.43 (−4.22, −0.63)	0.00796923	NR	Weak (insufficient data)
Wang *et al*. (2017) [38]	SBP, Chinese individuals with hypertension and hyperhomocysteinemia without history of stroke or MI	RCT	Supplement	NR (NA)	WMD	−7.85 (−9.43, −6.27)	0.00000000	95	Weak
Asbaghi *et al*. (2023) [48]	DBP, individuals with CVD, T2D, PCOS, HIV, menopause or alcohol/cigarette use	RCT	Supplement	41 589 (20 874)	WMD	−0.24 (−0.37, −0.11)	0.00029636	47.9	Suggestive
	*Baseline <80 mmHg*	RCT	Supplement	NR (NA)	WMD	−0.20 (−0.96, 0.54)	0.60120624	NR	Weak (insufficient data)
	*Baseline ≥80 mmHg*	RCT	Supplement	NR (NA)	WMD	−0.38 (−0.79, 0.01)	0.06260309	NR	Weak
	*Individuals with CVD*	RCT	Supplement	NR (NA)	WMD	−0.23 (−0.36, −0.09)	0.00084002	NR	Weak (insufficient data)
	*Individuals without CVD*	RCT	Supplement	NR (NA)	WMD	−0.52 (−1.42, 0.37)	0.25479870	NR	Weak (insufficient data)
	*Male*	RCT	Supplement	NR (NA)	WMD	−4.19 (−7.51, −0.86)	0.01351528	NR	Weak
	*Female*	RCT	Supplement	NR (NA)	WMD	−1.70 (−3.62, 0.21)	0.08186819	NR	Weak
	*BMI 18.5–24.9*	RCT	Supplement	NR (NA)	WMD	−0.30 (−0.47, −0.13)	0.00054254	NR	Weak (insufficient data)
	*BMI 25–29.9*	RCT	Supplement	NR (NA)	WMD	−0.15 (−0.38, 0.06)	0.18143045	NR	Weak (insufficient data)
Wang *et al*. (2017) [38]	DBP, Chinese individuals with hypertension and hyperhomocysteinemia without history of stroke or MI	RCT	Supplement	NR (NA)	WMD	−6.77 (−8.55, −5.00)	0.00000000	NR	Weak
Yi *et al*. (2014) [43]	Heart rate, individuals with CAD	RCT	Supplement	237 (NA)	MD	−0.39 (−2.89, 2.11)	0.75978739	0	Weak
Zhao *et al*. (2018) [30]	HbA1c, individuals with CVD, metabolic conditions, PCOS, or cervical intraepithelial neoplasia	RCT	Supplement	313 (NA)	WMD	−0.17 (−0.49, 0.16)	0.30525431	77.8	Weak
Sudchada *et al*. (2012) [49]	HbA1c, Iranian individuals with T2D	RCT	Supplement	142 (NA)	WMD	−0.37 (−1.10, 0.35)	0.31717703	83.8	Weak
Asbaghi *et al*. (2021) [50]	Fasting glucose, individuals with T2D, CVD, PCOS, endometrial hyperplasia or menopause	RCT	Supplement	34 593 (NA)	WMD	−2.17 (−3.69, −0.65)	0.00513950	81.5	Suggestive
	*Baseline <100 mg/dL*	RCT	Supplement	NR (NA)	WMD	−2.14 (−4.36, −0.06)	0.05107088	85.7	Weak
	*Baseline ≥100 mg/dL*	RCT	Supplement	NR (NA)	WMD	−4.06 (−7.83, −0.29)	0.03479215	71.9	Weak
	*Follow-up <12 weeks*	RCT	Supplement	NR (NA)	WMD	−5.32 (−9.11, −1.53)	0.00593701	86.5	Weak
	*Follow-up ≥12 weeks*	RCT	Supplement	NR (NA)	WMD	−0.79 (−1.81, 0.22)	0.12713014	48.6	Weak
	*FA dose <5 mg/d*	RCT	Supplement	NR (NA)	WMD	−1.40 (−3.23, 0.43)	0.13375601	84.9	Weak
	*FA dose ≥5 mg/d*	RCT	Supplement	NR (NA)	WMD	−3.58 (−6.62, −0.54)	0.02099036	78.3	Weak
	*Individuals without T2D*	RCT	Supplement	NR (NA)	WMD	−2.34 (−4.46, −0.22)	0.03051072	83.7	Weak
	*Individuals with T2D*	RCT	Supplement	NR (NA)	WMD	−4.87 (−10.15, 0.39)	0.07010473	73.6	Weak
	*Female*	RCT	Supplement	NR (NA)	WMD	−9.53 (−14.71, −4.35)	0.00031102	90.8	Weak (insufficient data)
	*Male*	RCT	Supplement	NR (NA)	WMD	−18.81 (−26.87, −10.74)	0.00000485	0	Weak (insufficient data)
Asbaghi *et al*. (2021) [50]	HOMA-IR, individuals with T2D, CVD, PCOS, endometrial hyperplasia or menopause	RCT	Supplement	644 (NA)	WMD	−0.40 (−0.70, −0.09)	0.01015542	80.9	Weak
	*Follow-up <12 weeks*	RCT	Supplement	227 (NA)	WMD	−0.62 (−0.64, −0.59)	0.00000000	0	Weak
	*Follow-up ≥12 weeks*	RCT	Supplement	390 (NA)	WMD	−0.31 (−0.83, 0.19)	0.23350736	83.7	Weak
	*FA dose <5 mg/d*	RCT	Supplement	179 (NA)	WMD	0.02 (−0.68, 0.73)	0.95565820	84	Weak
	*FA dose ≥5 mg/d*	RCT	Supplement	438 (NA)	WMD	−0.62 (−0.64, −0.60)	0.00000000	0	Weak
	*Individuals without T2DM*	RCT	Supplement	501 (NA)	WMD	−0.43 (−0.77, −0.08)	0.01456983	83.9	Weak
	*Individuals with T2DM*	RCT	Supplement	116 (NA)	WMD	−0.26 (−0.79, 0.27)	0.33629608	0	Weak
	*Female*	RCT	Supplement	381 (NA)	WMD	−0.38 (−0.82, 0.06)	0.09050739	85.8	Weak
	*Male*	RCT	Supplement	120 (NA)	WMD	−0.26 (−0.79, 0.27)	0.33629608	0	Weak
Akbari *et al*. (2018) [51]	Insulin, individuals with CVD, T2D, PCOS or metabolic conditions	RCT	Supplement	453 (NA)	SMD	−1.28 (−1.99, −0.56)	0.00045011	91.5	Weak
Asbaghi *et al*. (2021) [50]	Fasting insulin, individuals with CVD, T2D, PCOS, endometrial hyperplasia or menopause	RCT	Supplement	633 (NA)	WMD	−1.63 (−2.53, −0.73)	0.00038556	74.9	Weak
	*Follow-up <12 weeks*	RCT	Supplement	247 (NA)	WMD	−1.28 (−2.73, 0.16)	0.08252958	76	Weak
	*Follow-up ≥12 weeks*	RCT	Supplement	359 (NA)	WMD	−2.03 (−3.31, −0.75)	0.00188079	55.8	Weak
	*FA dose <5 mg/d*	RCT	Supplement	148 (NA)	WMD	−0.99 (−1.94, −0.04)	0.04109935	0.9	Weak
	*FA dose ≥5 mg/d*	RCT	Supplement	458 (NA)	WMD	−1.86 (−3.00, −0.71)	0.00145290	70.5	Weak
	*Individuals without T2D*	RCT	Supplement	490 (NA)	WMD	−1.96 (−2.92, −1.00)	0.00006290	65.3	Weak
	*Individuals with T2D*	RCT	Supplement	116 (NA)	WMD	0.02 (−1.45, 1.51)	0.97886931	0	Weak
	*Female*	RCT	Supplement	370 (NA)	WMD	−2.01 (−3.14, −0.88)	0.00048962	69	Weak
	*Male*	RCT	Supplement	116 (NA)	WMD	0.02 (−1.45, 1.51)	0.97886931	0	Weak
Tabrizi *et al*. (2018) [52]	Triglycerides, individuals with cardiometabolic conditions	RCT	Supplement	542 (NA)	SMD	0.10 (−0.42, 0.63)	0.70890037	88	Weak
Tabrizi *et al*. (2018) [52]	Total cholesterol, individuals with cardiometabolic conditions	RCT	Supplement	492 (NA)	SMD	0.06 (−0.31, 0.43)	0.75060795	74.9	Weak
Tabrizi *et al*. (2018) [52]	LDL-cholesterol, individuals with cardiometabolic conditions	RCT	Supplement	432 (NA)	SMD	−0.14 (−0.55, 0.28)	0.50848097	77.1	Weak
Tabrizi *et al*. (2018) [52]	HDL-cholesterol, individuals with cardiometabolic conditions	RCT	Supplement	492 (NA)	SMD	0.04 (−0.36, 0.44)	0.84461017	78.7	Weak
Tabrizi *et al*. (2018) [52]	VLDL-cholesterol, individuals with cardiometabolic conditions	RCT	Supplement	145 (NA)	SMD	0.08 (−0.24, 0.41)	0.62947811	0	Weak
Jafari *et al*. (2023) [53]	Body weight, individuals with T2D, PCOS or menopause	RCT	Supplement	378 (NA)	WMD	−0.16 (−0.47, 0.16)	0.31946614	40.4	Weak
Jafari *et al*. (2023) [53]	BMI, individuals with T2D, PCOS or menopause	RCT	Supplement	436 (NA)	WMD	−0.23 (−0.49, 0.03)	0.08294538	90.1	Weak
	*Homocysteine >15 µmol/L*	RCT	Supplement	NR (NA)	WMD	−0.17 (−0.33, 0.01)	0.04999579	NR	Weak
	*Individuals with PCOS*	RCT	Supplement	NR (NA)	WMD	−0.30 (−0.54, −0.06)	0.01428562	NR	Weak
Kazemi *et al*. (2022) [71]	PCOS, women aged 18–50 years	CS, CC	Dietary folate	9562 (1,161)	MD	−20.80 *µ*g/d (−42.65, 1.05)	0.06206762	37.5	Weak
**Dose-response analysis**									
Jayedi *et al*. (2019) [69]	CHD, individuals without history of CVD	PC	Dietary or total	212 284 (4022)	RR	0.79 (0.69, 0.89) for every 250 *µ*g/d increase	0.00024342	67	Suggestive
Wang *et al*. (2012) [32]	CHD, general population not otherwise stated	PC	Dietary folate	223 691 (2682)	RR	0.88 (0.82, 0.94) for every 200 *µ*g/d increase	0.06083747	27.4	Suggestive
Wang *et al*. (2012) [32]	CHD, general population not otherwise stated	PC	Plasma folate	14 533 (1936)	RR	0.95 (0.84, 1.00) for every 5 mmol/l increase	0.00028304	51.7	Weak
Chen *et al*. (2020) [68]	Stroke, individuals with CVD	PC	Dietary folate	253 511 (7429)	RR	0.94 (0.90, 0.98) for every 100 *µ*g/d increase	0.00439590	46.8	Suggestive

AMI – acute myocardial infarction, BMI – body mass index, BP – blood pressure, CAD – coronary artery disease, CC – case-control study, CHD – coronary heart disease, CIMT – carotid intima media thickness, CKD –coronary kidney disease, CS – cross-sectional study, CVCE – cardiovascular and cerebrovascular event, CVD – cardiovascular disease, CVT – cerebral venous thrombosis, DBP – diastolic blood pressure, DVT – deep vein thrombosis, EDD – endothelium-dependent dilation, ESRD – end-stage renal disease, FA – folic acid, FMD – flow-mediated dilation, GTN – glyceryl trinitrate, MACE – major adverse cardiovascular event, MD – mean difference, MI – myocardial infarction, NA – not applicable (continuous variable), NCC – nested case-control study, NR – not reported, PC – prospective cohort, PCOS – polycystic ovarian syndrome, PTE – pulmonary thromboembolism, PWV – pulse wave velocity, RCT – randomised controlled trial, RR – relative risk, RVO – retinal vein occlusion, SBP – systolic blood pressure, SMD – standardised mean difference, T2D – type 2 diabetes, VT – venous thrombosis, WMD – weighted mean difference

All six directional associations at the suggestive level of credibility showed an inverse association between folate status and cardiometabolic outcomes. A higher intake of dietary folate was associated with a lower risk of CHD in the general population and in individuals without history of CVD and a lower risk of stroke in individuals with hypertension, T2D, or history of stroke. Intake of FA supplements was effective in lowering SBP and DBP and fasting glucose in individuals with cardiometabolic conditions.

All five null associations at the suggestive level of credibility examined individuals with either existing or a history of CVD or ESRD. FA supplementation was not effective, compared to placebo, in reducing the risk of CVD, MACE, CHD, myocardial infarction, or total mortality in this population.

Three dose-response relationships were assessed to be of suggestive level of credibility. The risk of CHD decreased for every 200 *µ*g/d increase in dietary folate intake (RR = 0.88; 95% CI = 0.82, 0.94); the risk of CHD decreased for every 250 *µ*g/d increase in the intake of dietary or total folate; and the risk of stroke decreased for every 100 *µ*g/d increase in dietary folate intake (RR = 0.94; 95% CI = 0.90, 0.98).

## DISCUSSION

### Summary of findings

We identified 113 unique associations from 49 reviews that examined relationships between folate intake/status and risk of vascular and metabolic diseases. Most of the syntheses provided pooled risk estimates and all but one consisted of RCTs or prospective cohorts. While the meta-analyses of prospective cohorts primarily examined healthy individuals, all but one meta-analyses of RCTs were conducted in individuals with pre-existing cardiometabolic and other conditions. Overall risk of bias in these syntheses was low.

A relatively large volume of evidence was identified for composite CVD outcomes, CHD, and stroke. Evidence on other outcomes was limited to one or two syntheses for each category. Across the body of evidence, there was consistency in the direction and magnitude of the reported associations in each category of unique associations.

No association reached a convincing or highly suggestive level of credibility. Six directional associations and five null associations met the criteria for a suggestive level of credibility. In the general population, where the authors did not specifically state that the study population had comorbidities or other challenges, and in individuals without a history of CVD, a higher dietary intake of folate was associated with a reduced risk of CHD. In individuals with cardiometabolic conditions, a higher intake of dietary folate was associated with a lower risk of stroke. Three dose-response relationships, all at a suggestive level of credibility, supported an association between dietary folate intake and the risk of CHD and stroke. In individuals with cardiometabolic or other conditions, FA supplementation was effective in reducing blood pressure and fasting glucose levels. In individuals with either existing or a history of CVD or ESRD, FA supplementation was not effective in reducing the risk of CVD, MACE, CHD, MI, or all-cause mortality.

### Potential mechanisms of action

Several biological mechanisms by which folate may reduce the risk of CVD have been proposed. First, folate is involved in the remethylation of homocysteine to form methionine [74,75] with a consequent lowering of the plasma concentration of homocysteine [16,76–78]. Homocysteine is a known risk factor for atherosclerosis and cerebral small-vessel disease [79,80], as it reduces vasodilation and increases vascular resistance and increases smooth muscle cell proliferation [81]. Second, folate interacts with endothelial nitric oxide synthase and the endothelium-derived hyperpolarising factor to increase the production of nitric oxide [82–84], which plays anti-atherosclerotic, anti-inflammatory, and antioxidant functions in the endothelium [85–88]. Third, folate, through its role in one-carbon metabolism, contributes to normal DNA synthesis, DNA methylation, and DNA repair [89], which are important mechanisms in the prevention of many chronic diseases.

### Primary *vs*. secondary prevention and background folate status

The role of folate in cardiovascular health may be different in primary compared to secondary prevention settings [32,34,68,69]. Most of the component RCTs included in our reviews were designed to examine the effect of FA supplementation in individuals with pre-existing conditions on risk of adverse health effects and did not show conclusive evidence of a beneficial effect. In contrast, pooled estimates from prospective cohorts recruited otherwise healthy individuals, and all showed potential benefits of dietary folate in primary prevention of CHD [31,32,69]. Folate’s role in lowering homocysteine may be more beneficial in the early stages of vascular diseases compared to later stages marked by progression in arterial calcification [12,90]. It may also have more benefit among individuals with the highest homocysteine, rather than among those with a normal or high-normal homocysteine, or with frank folate deficiency [21,91]. Additionally, individuals in RCTs might have used other medications that interfere with folate, such as metformin. In trials of FA supplementation in reducing CVD risk in the general population, the baseline homocysteine concentration has been observed to be normal or high-normal, which may have resulted in a floor effect, that is a minimal level of plasma homocysteine below which FA has no further lowering effect [92]. Information on concomitant medications and their potential interaction with folate was not available in the included reviews.

Another potential explanation for the inconclusive evidence from RCTs may be the high dosage of FA used in the interventions [93]. Most of the meta-analyses included in our review reported wide ranges of FA dosage between 0.2 and 30 mg/d. Higher dosages may exceed recommended intake levels when combined with other dietary sources of folate [94]. Some authors suggest that FA supplementation at doses higher than physiologically relevant levels may be associated with a higher risk of other chronic conditions [68].

The protective effect of FA supplementation against stroke was comparable across areas with or without FA fortification; however, the effect size was the largest for countries without fortification [12,42]. This may be due to the plateauing effect in homocysteine lowering after FA exposure reaches adequacy [95]. Similar observations were made in another study, where FA fortification moderated the relationship between folate status and the risk of stroke [96]. Some authors further suggest that in countries where background population folate status is adequate, other nutrients may be more strongly associated with homocysteine status [97].

### Statistical *vs*. clinical significance

Contextualising statistically significant results into clinically meaningful findings is important, particularly when results are pooled from large datasets with high levels of heterogeneity. In our syntheses, we observed significant, but small effect sizes in several intermediate markers (*e.g.* SBP/DBP, glycaemic markers, endothelial markers) that may not imply clinically significant benefits. For example, a pooled estimate of WMD in SBP between a FA intervention and a control group was −1.11 mmHg (95% CI = −1.93, −0.28) [48]. While statistically significant and assessed to be at a suggestive level of credibility, the effect size did not reach what is commonly considered to be a clinically meaningful reduction (>3 mmHg) [98,99]. Likewise, the pooled estimate of WMD in fasting glucose was statistically significant (−2.17 mg/dL; 95% CI = −3.69, −0.65) [50] should be interpreted in the context of the baseline levels and other underlying risk factors. In a meta-analysis of 102 prospective studies, fasting blood glucose did not significantly reduce the risk of vascular disease in individuals with a history of diabetes or other risk factors [100].

### Equity and global health in the evidence on vascular and metabolic outcomes

Most of the syntheses consisted entirely or predominantly of intervention trials conducted in high income countries. We did not find any review with a focused scope on low- and middle-income countries (LMICs). A few reviews included studies from one or two middle-income countries in a larger pool of mostly European or North American studies. Only one review [42] included studies from several low-income countries in examining the relationship between FA supplementation and the risk of stroke. Although the authors reported risk estimates that were comparable to other reviews examining the same unique association, a subgroup analysis stratified by the socioeconomic background of the component studies might have enabled a more nuanced understanding of an association in LMICs.

The underrepresentation of LMICs in the current evidence is concerning considering the disproportionate burden of CVD and rising CVD mortality in these countries. Approximately 80% of global CVD deaths occur in LMICs [2,101] which also account for around 60% of global CVD DALYs lost [102]. The prevalence of CVD and CVD mortality in LMICs has been increasing in contrast to a declining trend in higher income countries [1,2,103], which may be attributed to lack of access to healthcare, limited treatment resources [104], and reduced health literacy and awareness. The severe imbalance between LMICs (2.8%) and high-income countries (81.1%) in contributing to global CVD research has been previously noted [102]. More evidence from LMICs examining modifiable risk factors of CVD, such as nutrition, will be valuable. For example, well-designed RCTs or large prospective cohort studies using validated folate exposure and outcome ascertainment will be add value to the current knowledge on the folate – CVD association. In particular, LMICs without mandatory FA fortification and/or with low folate status [105], such as China [106,107], may be informative populations in which to conduct future trials on CVD-related conditions.

### Limitations

This review has several limitations. First, baseline folate status of the individuals was not accounted for in most of the included syntheses, except for those that provided subgroup analyses by population-level FA fortification status. A predominant share of the syntheses pooled interventions of FA supplements, comparing FA to placebo in their effect on reducing cardiometabolic risk. However, the effect of FA supplement may be attenuated in individuals who maintain adequate folate status as compared to those with suboptimal or deficient folate status [108], and most of the syntheses did not adjust for the background folate status in their pooled estimates. Many trials were conducted in countries with varying forms of voluntary FA fortification [22], which may have played a role in the risk estimation [109]. Second, most of the syntheses examined RCTs, which ensure a high level of internal validity but may not be readily generalised to broad population settings. Related, most of these syntheses examined individuals with a diverse range of comorbidities, including pre-existing CVDs and likely with different levels of severity and complexity. These factors may limit the extrapolation of our findings directly to public health policies, and we therefore recommend a cautious interpretation of the evidence. Third, higher folate intakes are associated with plant-based diets, with reduced intake of other food groups (typically meat). The studies included in the reviews did not adjust for this, which may have contributed to the reported benefits of higher folate diets on study outcomes [110]. Fourth, most of the syntheses pooled a wide range of dosage and duration of FA supplementation. Only one review provided subgroup analyses by dosage and follow-up duration, some of which showed differential estimates. While a beneficial effect of FA doses higher than 5 mg/d was shown more consistently in this review, the overall evidence did not allow for detailed understanding of the effect of FA dose and duration on the risk of various outcomes. Fifth and lastly, we were constrained in resources to search two Chinese databases (CNKI and Wanfang) as originally planned [27]. Of the 46 meta-analyses included in this syntheses, 19 (41%) did not pool data from Chinese studies; 10 (22%) included Chinese studies (all published in or after 2015); and 17 (37%) did not specify the countries of data source. Considering the low folate status in the population [111], high prevalence of stroke [107], and lack of FA fortification in China, omission of more Chinese syntheses may have biased our findings toward the null. We reported associations between folic supplement use and the risk of stroke in countries with and without FA fortification [12]. However, the direction and magnitude of association were not very different. Future updates or similar reviews should incorporate searches from Chinese databases to improve comprehensiveness and reduce potential geographic bias.

## CONCLUSIONS

We critically appraised 113 unique associations between folate intake/status and the risk of vascular and metabolic diseases from existing systematic reviews. The available evidence consisted primarily of small-scale RCTs examining the effect of FA on secondary prevention. Studies conducted in low- and middle-income countries were substantially under-represented. A total of six directional associations (with CHD, stroke, blood pressure, and fasting glucose) and five null associations (with composite CVD, MACE, CHD, MI, and total mortality) reached a suggestive level of credibility. Among these, more large-scale studies are warranted to validate the relationship between dietary folate intake and CHD and stroke. Overall, the evidence landscape around folate and cardiometabolic diseases appears to be limited both in volume and scope.

## Additional material


Online Supplementary Document
