# Supplementary material for: Folate and global health review series, part 3: syntheses on cardiovascular, cerebrovascular, and metabolic diseases
Source: J Glob Health. 2026 May 11;16:04160. doi: 10.7189/jogh.16.04160 (PMC13159966; doi:10.7189/jogh.16.04160)
Supplement: Online Supplementary Document [file jogh-16-04160-s001.pdf]

Supplement to: Yoo S, Montazeri A, Bennett D, Bo Y, Chen P, Duthie S, Jensen N, Kaminga A, Lai J, Li X, MacFarlane A, Martinez H, McNulty H, Momoli F, Mossey P, Munger R, Parajuli RP, Kent M, Rubini M, Senekal M, Sikora L, Stintzi A, Theodoratou E, Wang H, Yaktine A, Little J. Folate and global health review series, part 3: syntheses on cardiovascular, cerebrovascular, and metabolic diseases. J Glob Health. 2026;16:04160.

Supplementary Table 1a. Characteristics of the evidence syntheses of randomized controlled trials examining the association between folate intake/status and cardio-, cerebrovascular and metabolic diseases (chronological)

First author (year)	Synthesis type	Study population	Folate exposure	Outcome	No. of studies (Design)	Total (Case)	Country	Comparators
Eikelboom (1999)	SR	Individuals with MI, unstable angina, arterial vascular disease, stroke, vascular disease	Supplement (0.8-5 mg/d vs placebo)	Treatment of vascular disease	4 (RCT)	19,000 (NR)	Norway, UK, Canada, Australia, US	Placebo
Bazzano (2006)	MA	Individuals with CHD or ESRD or stroke or diabetes	Supplement (0.5-15 mg/d for 0.5-5 years	CVD events	11 (RCT)	14,440 (2,706)	US, Australia, New Zealand, Canada, European countries	Placebo
				CHD	11 (RCT)	16,877 (1,862)		Placebo
				Stroke	8 (RCT)	13,806 (722)		Placebo
				Total mortality	10 (RCT)	14,995 (1,821)		Placebo
De Bree (2007)	MA	Individuals with CVD or high cholesterol or slightly elevated Hcy	Supplement (400-10,000 µg/d for 4-52 weeks)	%FMD	10 (RCT)	712 (355)	NR	Placebo
Wang X (2007)	MA	Individuals with stroke, CHD, ESRD, esophageal dysplasia	Supplement (0.5-15 mg/d for 24-72 months)	Stroke	8 (RCT)	16,841 (NR)	NR	Placebo
Van Hattum (2007)	SR	Individuals who underwent angioplasty with or without stenting for at least one coronary stenosis of ≥ 50%, individuals awaiting coronary stenting; individuals with stable coronary artery disease	Supplement	Coronary artery disease	3 (RCT)	1,434 (NR)	NR	Placebo
Bartlett HE (2008)	SR	Individuals with T2DM with or without hyperhomocysteinemia	Supplement (5-10 mg/d for 2 weeks – 6 months)	Endothelia-dependent vasodilation or endothelial function	3 (RCT)	86 (NR)	NR	Placebo
Heinz (2009)	MA	Individuals with ESRD undergoing dialysis	Supplement (5-15 mg/d for 12-29 months)	CVD	3 (RCT)	381 (138)	Italy, Brazil, US	Placebo
Khandanpour (2009)	SR	Individuals with peripheral arterial disease	Supplement (5-10 mg/d for 2-8 weeks)	ABPI change, pain-free walking distance, changes in inflammatory markers, changes in cholesterol	8 (RCT)	410 (NR)	Sweden, Australia, Hong Kong, Czech, Germany, Spain, Austria	Placebo
McRae (2009)	MA	Individuals with	Supplement (5-10	%FMD change	8 (RCT)	372 (NA)	NR	Placebo

		hypertension	mg/d for 2-16 weeks)	Systolic BP change	8 (RCT)	293 (NA)	NR	Placebo
				Diastolic BP change	8 (RCT)	293 (NA)	NR	Placebo
Mente (2009)	MA	General population	Supplement	Coronary heart disease	16 (RCT)	NR (NR)	NR	Placebo
Lee (2010)	MA	Individuals with AMI, CHD, ESRD	Supplement (0.5-15mg/d for 12-43 months)	Stroke	4 (RCT)	1,279 (50)	Netherlands, Italy, Australia, New Zealand	Placebo
Pase (2011)	SR	Individuals with chronic renal failure or PAD or male with normal/high BP	Supplement (5-15 mg/d for 3-173 weeks, 400 µg/d FA + 400 µg/d MTHF for 16 weeks)	Arterial stiffness	3 (RCT)	489 (NR)	NR	Nonactive control
Qin X (2011)	MA	Individuals with ESRD or ACKD	Supplement (5-15 mg/d for 12-43 months, 30 mg/wk for 24 months)	Fatal or non-fatal CV events	3 (RCT)	582 (177)	Australia, New Zealand, Brazil, Europe	Placebo
Zhou YH (2011)	MA	Individuals with diabetic nephropathy, stroke, CHD, ACKD/ESRD, CRF, MI, hemodialysis	Supplement (0.5-40 mg/d for 8.3-87.6 months)	Major CV event	12 (RCT)	38,015 (8,238)	NR	Placebo
				Stroke	12 (RCT)	42,960 (2,001)		Placebo
				MI	11 (RCT)	39,923 (2,917)		Placebo
				All-cause mortality	14 (RCT)	44,340 (6,314)		Placebo
				Vascular death	10 (RCT)	27,342 (NR)		Placebo
				Non-vascular death	8 (RCT)	21,314 (NR)		Placebo
				Revascularization	10 (RCT)	38,068 (NR)		Placebo
				Coronary syndrome	5 (RCT)	19,050 (3,148)		Placebo
Qin X (2012)	MA	Individuals with ESRD or ACKD or renal transplants or CVD risk or CHD or general population with ≥ 8.5 umol/L Hcy	Supplement (0.8-15 mg/d for 3-42 months)	IMT (intima media thickness) change	4 (RCT)	1,459 (NA)	Australia, New Zealand, Brazil, Spain, Iran, Greece, Netherlands	Placebo
Pan (2012)	MA	Individuals on hemodialysis or peritoneal dialysis	Supplement (5-15 mg/d for 1-3.6 years)	CVD incidence	5 (RCT)	1,180 (348)	NR	Placebo
Huo (2012)	MA	Individuals with pre-existing CHD, ESRD, or ACKD	Supplement (0.5 mg/d for 41 months or 15 mg/d for 43 months)	Stroke	2 (RCT)	908 (46)	NR	Placebo
Sudchada P (2012)	MA	Individuals with T2DM	FA supplement (5 mg/d for 4-8 weeks)	HbA1c	3 (RCT)	142 (NR)	Iran	Placebo
Qin X (2013)	MA	Individuals with ESRD or ACKD	Supplement (5-15 mg/d for 12-43 months, 30 mg/wk for 24 months)	Fatal, non-fatal CV events	4 (RCT)	582 (177)	Australia, New Zealand, Brazil, Europe	Placebo

Myung (2013)	MA	General population	Supplement (0.5-15 mg/d for 1-3.6 years)	Major CV events	4 (RCT)	2,871 (282)	Netherlands, Italy, Australia, New Zealand	Placebo
Zhang C (2013)	MA	Individuals with chronic renal failure or stable coronary artery disease, or colorectal adenomas	Supplement (0.5-1.5 mg/d for 3.2-7 years)	Stroke	3 (RCT)	1,929 (NR)	NR	Placebo
Yi (2014)	MA	Individuals with CAD	Supplement (5 mg/d for 4-16 wks)	Endothelial function	6 (RCT)	1,796 (NA)	NR	Placebo
				Mean FMD	4 (RCT)	287 (NA)	NR	Placebo
				Mean EDD	4 (RCT)	237 (NA)	NR	Placebo
				GTN diameter change	3 (RCT)	187 (NA)	NR	Placebo
				Baseline hyperemic flow	3 (RCT)	187 (NA)	NR	Placebo
				Peak hyperemic flow	3 (RCT)	187 (NA)	NR	Placebo
				Heart rate	4 (RCT)	237 (NA)	NR	Placebo
				Systolic BP	4 (RCT)	237 (NA)	NR	Placebo
				Diastolic BP	4 (RCT)	237 (NA)	NR	Placebo
Liu Y (2014)	MA	Individuals with CAD	Supplement (400-10,000 µg/d for 4-16 wks)	FMD (flow-mediated dilation)	11 (RCT)	611 (NA)	NR	Placebo
Dong (2015)	MA	General population	FA supplement	Stroke	2 (RCT)	NR (NR)	NR	Placebo
Zeng (2015)	MA	Fortification Individuals with cardiometabolic/ renal conditions	FA supplement (2.5-40 mg/d for 32-87 months)	Stroke	4 (RCT)	8,051 (258)	Australia, New Zealand, US, Canada	Placebo
		Partial fortification Individuals with cardiometabolic/ renal conditions	FA supplement (0.02-2.5 mg/d for 24-60 months)	Stroke	3 (RCT)	17,366 (1,306)	Canada, US, UK, Brazil, others	Placebo
		No fortification Individuals with cardiometabolic/ renal conditions	FA supplement (0.2-2 mg/for 24-78 months)	Stroke	7 (RCT)	24,260 (846)	China, Netherlands, Norway, Germany, France, UK	Placebo
Zhang C (2014)	MA	Individuals with CHD or ESRD or colorectal adenomas	Supplement (0.5-25 mg/d for 1-7 years)	Major CV events	7 (RCT)	4,331 (NR)	NR	Placebo
Li Y (2016)	MA	General population aged 49-69 years	Supplement (3.2 years)	Stroke	5 (RCT)	NR (773)	Canada, US, Brazil, Scotland, Norway, China, Netherlands, Germany, France, Italy, Australia, New Zealand	Placebo

				CHD	7 (RCT)	NR (689)	Canada, US, UK, Germany, Netherlands, Philippines, Australia, New Zealand, Norway, Brazil, China, Scotland, France, India, Italy, Switzerland	Placebo
				CVD	8 (RCT)	NR (1,627)	Canada, US, UK, Norway, Brazil, Netherlands, Scotland, France, Australia, New Zealand, China, Germany, Italy	Placebo
Schwingshackl (2017)	MA	Individuals without history of CVD, renal conditions, cancer, GI, neurological conditions	Supplement	All-cause mortality	2 (RCT)	NR (NR)	NR	Placebo
				CVD incidence	3 (RCT)	NR (NR)	NR	Placebo
Wang WW (2017)	MA	Individuals with diagnosis of hypertension and hyperhomocysteinemia without history of stroke or MI, China	Supplement (0.4-10 mg/d FA + antihypertensives for 8 wks-18 months)	Systolic BP	49 (RCT)	5,707 (NA)	China	Antihypertensives
				Diastolic BP	49 (RCT)	5,707 (NA)		
				CVCE	22 (RCT)	2,057 (300)		
Zhao M (2017)	MA	Individuals with CHD or hypertension	FA supplement (0.5-0.8 mg/d for 42-54 months)	Stroke	2 (RCT)	21,295 (657)	NR	Placebo
Hsu CY (2018)	MA	Individuals with pre-existing cardiovascular conditions and mostly living in countries without mandatory FA fortification	FA supplement (alone or with minimal (≤ 0.05 mg/d) cyanocobalamin)	Stroke	8 (RCT)	28,450 (827)	China, Netherlands, Australia, New Zealand, Norway, Italy, Germany, UK, France, Austria, Brazil, Belgium, India, Moldova, Malaysia, Pakistan, Philippines, Portugal, Georgia, Serbia, Singapore, Sri Lanka	Placebo
Tabrizi R (2018)	MA	Individuals with T2DM, metabolic syndrome, obesity, CAD, PCOS, or diabetic neuropathy	FA supplement (2.5-5 mg/d for 4-12 weeks)	Systolic BP	6 (RCT)	262 (NA)	Australia, Italy, UK, Iran, Germany	Placebo
				Diastolic BP	6 (RCT)	262 (NA)		Placebo
			FA supplement (2.5-10 mg/d for 2-12 weeks)	Triglycerides	11 (RCT)	542 (NA)	Australia, Iran, Turkey, Italy, Canada, UK, Taiwan	Placebo
				Total cholesterol	10 (RCT)	492 (NA)	Australia, Iran, Turkey, Italy, Canada, UK, Taiwan	Placebo

				LDL-cholesterol	9 (RCT)	432 (NA)	Australia, Iran, Turkey, Canada, UK, Taiwan	Placebo
				HDL-cholesterol	10 (RCT)	492 (NA)	Australia, Iran, Turkey, Italy, Canada, UK, Taiwan	Placebo
			FA supplement (2.5-5 mg/d for 8-12 weeks)	VLDL-cholesterol	3 (RCT)	145 (NA)	Iran, Turkey	Placebo
Zhao JV (2018)	MA	Individuals with obesity, stable CHD, cervical intraepithelial neoplasia, PCOS, hypertension, microalbuminuria, metabolic syndrome	FA supplement (0.15-10 mg/d for 2 weeks – 4.5 years)	Fasting glucose	15 (RCT)	16,768 (NA)	Iran, China, Turkey, Israel, Italy, Canada, UK	Placebo
			FA supplement (0.15-5 mg/d for 4 weeks – 6 months)	HbA1c	4 (RCT)	313 (NA)	Iran, China, Israel, UK	Placebo
			FA supplement (0.35-15 mg/d for 3 weeks – 12 months)	HOMA-IR	9 (RCT)	435 (NA)	Iran, Italy, Turkey,	Placebo
			FA supplement (1-15 mg/d for 3 weeks – 12 months)	Insulin	8 (RCT)	380 (NA)	Iran, Italy	Placebo
Akbari (2018)	MA	Individuals with metabolic diseases	FA supplement (1-10 mg/d for 4-12 weeks)	Insulin	8 (RCT)	453 (226)	Iran, Italy, UK, Australia, New Zealand, Taiwan, Turkey, Canada	Placebo
				HOMA-IR	9 (RCT)	499 (249)		Placebo
				Fasting glucose	10 (RCT)	511 (254)		Placebo
				HbA1c	7 (RCT)	309 (158)		Placebo
Wang Y (2019)	MA	Individuals with CVD	Supplement (0.5-5 mg/d for 12-60 months)	CHD	4 (RCT)	24,393 (NR)	Netherlands, Norway, China	Placebo
				Stroke	4 (RCT)	24,393 (NR)		
Lind MV (2019)	MA	Individuals with obesity, metabolic syndrome, CHD, CAD, T2DM, hypertension, AMI, PCOS, or cervical intraepithelial neoplasia	FA supplement (0.4-15 mg/d for 2-26.1 weeks)	Fasting glucose	23 (RCT)	17,242 (NA)	Iran, Italy, Taiwan, Norway, Japan, UK, Brazil, Australia, China, Netherlands, Canada	Placebo
			FA supplement (1-15 mg/d for 3-26.1 weeks)	HOMA-IR	9 (RCT)	431 (NA)	Iran, Italy, Taiwan	Placebo
			FA supplement (1-15 mg/d for 3-26.1 weeks)	Fasting insulin	9 (RCT)	431 (NA)	Iran, Italy, Taiwan	Placebo
			FA supplement (0.25-5 mg/d for 4-26.1 weeks)	HbA1c	5 (RCT)	211 (NA)	Norway, Iran, Australia, Netherlands	Placebo

Jenkins (2021)	MA	Individuals with CVD	Supplement (0.5-15 mg/d for 1-10 years, 800 µg/d for 3 years)	Total CVD<br>Total CHD<br>MI<br>Stroke<br>CVD mortality<br>MI mortality<br>Stroke mortality<br>All-cause morality	5 (RCT)<br>2 (RCT)<br>6 (RCT)<br>7 (RCT)<br>5 (RCT)<br>2 (RCT)<br>2 (RCT)<br>10 (RCT)	21,567 (960)<br>2,197 (77)<br>24,210 (140)<br>24,525 (707)<br>22,468 (201)<br>20,985 (13)<br>20,985 (29)<br>25,580 (895)	China, US, UK, Denmark, Brazil, Netherlands, Canada, Australia, New Zealand, Italy	Placebo
Asbaghi O (2021)	MA	Individuals with T2DM, CAD, PCOS, obesity, AMI, hypertension, metabolic syndrome, endometrial hyperplasia, or menopause	FA supplement (0.25-10 mg/d for 2-234 weeks)	Fasting glucose	27 (RCT)	34,641 (NA)	NR	Placebo
			FA supplement (025-5 mg/d for 4-12 weeks)	HbA1c	4 (RCT)	170 (NA)		Placebo
			FA supplement (0.4-15 mg/d for 3-25 weeks)	Fasting insulin	12 (RCT)	633 (NA)		Placebo
			FA supplement (0.348-15 mg/d for 3-25 weeks)	HOMA-IR	12 (RCT)	644 (NA)		Placebo
Saz-Lara (2022)	MA	Individuals with or without T2DM	Supplement (5mg/d for 4 wks)	Arterial stiffness (pulse wave velocity)	2 (RCT)	50 (NA)	UK	Placebo
Asbaghi (2023)	MA	Individuals with cardiovascular conditions, T2DM, PCOS, HIV, postmenopausal, or alcohol/cigarette use	Supplement	Systolic BP	26 (RCT)	41,633 (20,896)	Canada, UK, Italy, Australia, US, Belgium, Korea, China, Iran, Germany, Brazil, Czech Republic	Placebo
				Diastolic BP	24 (RCT)	41,589 (20,874)	Canada, UK, Italy, Australia, US, Belgium, Korea, China, Iran, Brazil, Czech Republic	Placebo
Bokayeva (2023)	MA	General population	Supplement (5-15 mg/d for 4-188 wks)	Central pulse wave velocity	4 (RCT)	421 (NA)	Australia, New Zealand, UK	Placebo
			Supplement (400 µg/d-10 mg/d for 2-17 wks)	FMD	14 (RCT)	601 (NA)	Australia, UK, Canada, Brazil, US, Belgium, Turkey, Netherlands	Placebo
			Supplement (5-15 mg/d for 4-206 wks)	Peripheral pulse wave velocity	2 (RCT)	553 (NA)	Australia, New Zealand	Placebo
Jafari A (2023)	MA	Individuals with obesity, menopause, PCOS, diabetes or cervical intraepithelial neoplasia	FA supplement (1.5-15 mg/d for 3-24 weeks)	Body weight	7 (RCT)	378 (NA)	Taiwan, Italy, Iran	Placebo
			FA supplement (1.5-7.5 mg/d for 3-12 weeks)	BMI	9 (RCT)	436 (NA)	UK, Taiwan, Italy, Iran	Placebo

ACKD: advanced chronic kidney disease; AMI: acute myocardial infarction; BMI: body mass index; BP: blood pressure; CAD: coronary artery disease; CHD: coronary heart disease; CC: case-control study; CIMT: carotid intima media thickness; CVCE: cardiovascular and cerebrovascular event; CVD: cardiovascular disease; CVT: cerebral venous thrombosis; DBP: diastolic blood pressure; DVT: deep vein thrombosis; EDD: endothelium-dependent dilation; ESRD: end-stage renal disease; FMD: flow-mediated dilation; GTN: glyceryl trinitrate; MI: myocardial infarction; NA: not applicable (continuous variable); NCC: nested case-control; NR: not reported; PC: prospective cohort; PCOS: polycystic ovarian syndrome; PTE: pulmonary thromboembolism; PWV: pulse wave velocity; RCT: randomized controlled trial; RVO: retinal vein occlusion; SBP: systolic blood pressure; T2D: type 2 diabetes; VT: venous thrombosis.

Supplementary Table 1b. Characteristics of the evidence syntheses of observational studies examining the association between folate intake/status and cardio-, cerebrovascular and metabolic diseases (chronological)

First author (year)	Synthesis type	Study population	Folate exposure	Outcome	No. of studies (Design)	Total (Case)	Country	Comparators
Eikelboom (1999)	SR	General population	Serum folate	CVD	5 NCC, 2 CC	NR (NR)	Canada, US, 9 European countries	High vs low
Mente (2009)	MA	General population	Total intake	Coronary outcomes	2 (PC)	308,012 (NR)	NR	High vs low
			Dietary intake	Coronary outcomes	4 (PC)	104,307 (NR)	NR	High vs low
Zhou K (2012)	MA	Individuals matched on age, sex, and major acquired risk factors	Plasma folate	VT	12 (CC)	4,197 (1,937)	NR	Case vs control
			Plasma folate	DVT/PTE	5 (CC)	2,632 (1,250)	NR	Case vs control
			Plasma folate	CVT	3 (CC)	622 (226)	NR	Case vs control
			Plasma folate	RVO	3 (CC)	752 (398)	NR	Case vs control
Wang ZM (2012)	MA	General population	Dietary intake	CHD	7 (PC)	223,691 (2,682)	US, Finland, Germany, Japan, Netherlands, Sweden	High vs low
		General population	Plasma/serum (mmol/L)	CHD	8 (PC)	14,533 (1,936)	US, Canada, Australia, Netherlands, Finland, Sweden	High vs low
Foroughi (2013)	SR	NR	Dietary<br>Plasma folate<br>Plasma folate	Hypertension<br>Stroke<br>CVT	1 (PC)<br>1 (NCC)<br>1 (CC)	94,334 (NR)	US, Sweden, Mexico	High vs low
Colapinto (2016)	SR	General population	Blood concentration	CVD	10 (NR)	16,159 (NR)	NR	High vs low
Jayedi (2019)	MA	Individuals with a history of CVD	Dietary intake	CHD	7 (PC)	232,549 (3,952)	Japan, Netherlands, Germany Sweden, US, Finland	High vs low
			Total intake		3 (PC)	NR (NR)		
			Dietary/total intake		9 (PC)	235,284 (4,281)		
Chen L (2020)	MA	General population	Dietary folate	Stroke	9 PC, 1 NCC	255,458 (7,895)	US, Netherlands, Finland, Sweden, China	High vs low
Kazemi (2022)	MA	Women aged 18-50 years	Dietary folate	PCOS	5 CS, 4 CC	9,562 (1,161)	Canada, US, Turkey, Iran, Poland, China	Case vs control

ACKD: advanced chronic kidney disease; AMI: acute myocardial infarction; BMI: body mass index; BP: blood pressure; CAD: coronary artery disease; CHD: coronary heart disease; CC: case-control study; CIMT: carotid intima media thickness; CS: cross-sectional study; CVCE: cardiovascular and cerebrovascular event; CVD: cardiovascular disease; CVT: cerebral venous thrombosis; DBP: diastolic blood pressure; DVT: deep vein thrombosis; EDD: endothelium-dependent dilation; ESRD: end-stage renal disease; FMD: flow-mediated dilation; GTN: glyceryl trinitrate; MI: myocardial infarction; NA: not applicable (continuous variable); NCC: nested case-control; NR: not reported; PC: prospective cohort; PCOS: polycystic ovarian syndrome; PTE: pulmonary thromboembolism; PWV: pulse wave velocity; RCT: randomized controlled trial; RVO: retinal vein occlusion; SBP: systolic blood pressure; T2D: type 2 diabetes; VT: venous thrombosis.

Supplementary Table 2. Summary of the meta-analyses reporting the association of folate intake/status with the risk of vascular or metabolic diseases

Author (year)	Outcome	Study population/ subgroup	No. studies (design)	Dose, duration, follow-up (mean, range)	No. total (case)	Comparator	Summary effect ()	I^2	P_{Egger}
**CLINICAL EVENTS**									
**Cardiovascular disease (CVD) – Supplement**									
Schwingshackl (2017)	CVD	Generally healthy population	3 (RCT)	0.4-5 mg/d for 12-54 months	NR (NR)	Placebo	**RR=0.81 (0.70, 0.94)**	0%	NR
Li Y (2016)	CVD	General population	8 (RCT)	NR	NR (1,627)	Placebo	**RR=0.90 (0.82, 0.99)**	30% (p=0.19)	> 0.05
Bazzano (2006)	CV events	Individuals with history of CVD or ESRD	11 (RCT)	0.5 mg/d-15 g/d for 0.5-5 years	14,440 (2,706)	Placebo	RR=0.95 (0.88, 1.03)	p=0.33	No*
Qin X (2011)	CV events	Individuals with kidney disease	3 (RCT)	5-30 mg/d for 12-43 months	582 (177)	Placebo	RR=0.83 (0.65, 1.06)	NR	NR
Qin X (2013)	CV events	Individuals with kidney disease	4 (RCT)	5-30 mg/d for 12-43 months	582 (177)	Placebo	RR=0.83 (0.65, 1.06)	NR	NR
Pan (2012)	CVD	Individuals with CKD (with or without dialysis)	5 (RCT)	5-15 mg/d for 12-43 months	1,180 (348)	Placebo	RR=0.88 (0.75, 1.03)	0% (p=0.48)	NR
Heinz (2009)	CVD	Individuals with ESRD undergoing dialysis	3 (RCT)	5-15 mg/d for 12-29 months	381 (138)	Placebo	**RR=0.73 (0.56, 0.94)**	NR	NR
Jenkins (2021)	CVD	Individuals with CVD, ESRD, or history of colorectal adenomas	5 (RCT)	0.8-15 mg/d for 12-54 months	21,567 (960)	Placebo	**RR=0.83 (0.73, 0.93)**	0% (p<0.01)	NR
Wang WW (2017)	CVCE	Chinese individuals with hypertension and hyperhomocysteinemia without history of stroke or MI	22 (RCT)	0.4-10 mg/d for 8 weeks-18 months	2,057 (300)	Anti-hypertensives only	**RR=0.30 (0.21, 0.43)**	43% (p=0.02)	<0.05
Major CV events									
Myung (2013)	Major CV events	General population	4 (RCT)	0.5-15 mg/d for 12-43 months	2,871 (282)	Placebo	RR=1.02 (0.84, 1.23)	47%	NR
Zhou YH (2011)	Major CV events	Individuals with CVD or ESRD	12 (RCT)	0.5-5 mg/d for 24-87.6 months	38,015 (8,238)	Placebo	RR=0.98 (0.93, 1.04)	31.8% (p=0.14)	NR
Zhang C (2014)	Major CV events	Individuals with CHD, ESRD, or colorectal adenomas	7 (RCT)	0.5-25 mg/d for 12-84 months	4,331 (NR)	Placebo	RR=1.02 (0.96, 1.08)	p=0.48	NR
**Coronary Heart Disease (CHD) – Dietary intake**									
Mente (2009)	Coronary outcomes	General population	4 (PC)	NR	104,307 (NR)	High vs low	RR=0.62 (0.50, 0.79)	NR	NR
Wang ZM (2012)	CHD	General population	7 (PC)	4.6-14 years	223,691 (2,682)	High vs low	**RR=0.69 (0.60, 0.80)**	0% (p=0.47)	0.95
Jayedi (2019)	CHD	Individuals without history of CVD	7 (PC)	NR	232,549 (3,952)	High vs low	**RR=0.68 (0.53, 0.84)**	63% (p=0.01)	NR
**Coronary Heart Disease (CHD) – Supplement**									
Mente	Coronary	General population	16 (RCT)	NR	NR (NR)	Placebo	RR=0.99 (0.91, 1.06)	NR	NR

(2009)	outcomes								
Li Y (2016)	CHD	General population	5 (RCT)	NR	NR (689)	Placebo	RR=1.13 (0.98, 1.31)	0% (p=0.66)	>0.05
Bazzano (2006)	CHD	Individuals with history of CVD or ESRD	11 (RCT)	0.5 mg/d-15 g/d for 6-60 months	16,877 (1,862)	Placebo	RR=1.04 (0.92, 1.17)	p=0.15	No*
Wang Y (2019)	CHD	Individuals with CVD	4 (RCT)	0.5-5 mg/d for 12-60 months	24,393 (NR)	Placebo	RR=1.08 (0.92, 1.27)	0% (p=0.89)	NR
Zhou YH (2011)	Coronary syndrome	Individuals with CVD or ESRD	5 (RCT)	0.5-5 mg/d for 8.3-72 months	19,050 (3,148)	Placebo	RR=1.06 (0.97, 1.15)	10.4% (p=0.35)	NR
Jenkins (2021)	CHD	Individuals with CVD, ESRD or history of colorectal adenoma	2 (RCT)	5-15 mg/d for 20-43 months	2,197 (77)	Placebo	RR=1.47 (0.95, 2.28)	0% (p=0.08)	NR
**Coronary Heart Disease (CHD) – Total intake**									
Mente (2009)	Coronary outcomes	General population	2 (PC)	NR	308,012 (NR)	High vs low	**RR=0.68 (0.57, 0.79)**	NR	NA
Jayedi (2019)	CHD	Individuals without history of CVD	3 (PC)	NR	NR (NR)	High vs low	**RR=0.73 (0.59, 0.87)**	0% (p=0.62)	NA
**Coronary Heart Disease (CHD) – Dietary or Total intake**									
Jayedi (2019)	CHD	Individuals without history of CVD	9 (PC)	8-16.5 years	235,284 (4,281)	High vs low	RR=0.69 (0.57, 0.81)	54% (p=0.03)	0.72
		US	2 (PC)	14-16.5 years	93,602 (2,408)	High vs low	RR=0.73 (0.64, 0.83)	0% (p=0.49)	NA
		Europe	5 (PC)	4.6-13 years	42,149 (1,198)	High vs low	RR=0.74 (0.47, 1.01)	67% (p=0.02)	NA
		Asia	2 (PC)	11.5-14 years	99,533 (675)	High vs low	RR=0.67 (0.48, 0.87)	0% (p=0.60)	NA
**Coronary Heart Disease (CHD) – Plasma folate**									
Wang ZM (2012)	CHD	General population	8 (PC)	3.3-29 years	14,533 (1,936)	High vs low	RR=0.74 (0.53, 1.02)	64.9% (p=0.002)	0.26
**Stroke – Dietary intake**									
Chen L (2020)	Stroke	Individuals with hypertension, T2DM or a history of stroke	9 PC, 1 NCC	4.2-19 years	255,458 (7,895)	High vs low	**RR=0.85 (0.78, 0.94)**	11.5%	0.76
**Stroke - Supplement**									
Li Y (2016)	Stroke	General population	5 (RCT)	NR	NR (773)	Placebo	**RR=0.79 (0.69, 0.92)**	11% (p=0.35)	> 0.05
Bazzano (2006)	Stroke	Individuals with history of CVD or ESRD	8 (RCT)	0.5mg/d-15g/d for 0.5-5 years	13,806 (722)	Placebo	RR=0.86 (0.71, 1.04)	p=0.27	No*
Zhao M (2017)	Stroke	Individuals with CVD	2 (RCT)	0.5-0.8 mg/d for 42-54 months	21,295 (657)	Placebo	RR=0.80 (0.70, 0.93)	0% (p=0.975)	NR
Wang Y (2019)	Stroke	Individuals with CVD	4 (RCT)	0.5-5 mg/d for 12-60 months	24,393 (NR)	Placebo	RR=0.80 (0.70, 0.93)	0% (p=0.75)	NR
Wang, X (2007)	Stroke	Individuals with CVD, ESRD or esophageal dysplasia	8 (RCT)	0.5-15 mg/d for 24-72 months	16,841 (NR)	Placebo	RR=0.82 (0.68, 1.00)	NR	NR
		With FA fortification	3 (RCT)	2.5-15 mg/d for 24-43 months	4,505 (353)	Placebo	RR=0.89 (0.55, 1.42)	NR	NR
		Without FA fortification	5 (RCT)	0.5-15 mg/d for 29-72 months	12,336 (425)	Placebo	RR=0.75 (0.62, 0.91)	NR	NR
Lee (2010)	Stroke	Individuals with CVD, ESRD or esophageal dysplasia	4 (RCT)	0.5-15 mg/d for 12-43 months	1,279 (50)	Placebo	RR=0.57 (0.33, 1.01)	NR	NR
Zhou YH	Stroke	Individuals with CVD or ESRD	12 (RCT)	0.5-5 mg/d for 12-72	42,960 (2,001)	Placebo	RR=0.89 (0.78, 1.01)	31.6% (p=0.14)	NR

(2011)				months					
Huo (2012)	Stroke	Individuals with CVD or ESRD/ACKD	2 (RCT)	0.5-15 mg/d for 42-43 months	908 (46)	Placebo	RR=0.53 (0.30, 0.96)	NR	NR
Zhang C (2013)	Stroke	Individuals with CVD, ESRD, or colorectal adenomas	3 (RCT)	0.5-15 mg/d for 38-84 months	1,929 (NR)	Placebo	RR=0.79 (0.68, 0.92)	NR	NR
Jenkins (2021)	Stroke	Individuals with CVD, ESRD or history of colorectal adenoma	7 (RCT)	0.5-5 mg/d for 12-120 months	24,525 (707)	Placebo	**RR=0.79 (0.69, 0.92)**	0% (p<0.01)	NR
Dong (2015)	Stroke	Individuals with colorectal adenomas or ESRD	2 RCT	NR	NR (NR)	Placebo	RR=0.79 (0.62, 1.01)	NR	>0.05
Zeng (2015)	Stroke	Individuals with cardiometabolic or renal conditions in countries with fortification	4 (RCT)	2.5-40 mg/d for 32-87 months	8,051 (258)	Placebo	RR=0.94 (0.58, 1.54)	61% (p=0.05)	NR
		Individuals with cardiometabolic or renal conditions in countries with partial fortification	3 (RCT)	0.02-2.5 mg/d for 24-60 months	17,366 (1,306)	Placebo	RR=0.91 (0.82, 1.01)	47% (p=0.15)	NR
		Individuals with cardiometabolic or renal conditions in countries with no fortification	7 (RCT)	0.2-2 mg/d for 24-78 months	24,260 (846)	Placebo	RR=0.88 (0.77, 1.00)	1% (p=0.42)	NR
Hsu CY (2018)	Stroke	Individuals CVD, ESRD, or esophageal dysplasia mostly living in countries without mandatory FA fortification	8 (RCT)	NR	28,450 (827)	Placebo	**RR=0.75 (0.66, 0.86)**	0% (p=0.77)	No*
**Thrombosis – Plasma folate**									
Zhou K (2012)	VT	Individuals with VT matched on age, sex, and major acquired risk factors	12 (CC)	NA	4,197 (1,937)		**SMD= -0.55 (-0.75, -0.36)**	88% (p<0.00001)	NR
	DVT/ PTE		5 (CC)	NA	2,632 (1,250)		**SMD= -0.42 (-0.66, -0.17)**	87% (p<0.00001)	NR
	CVT		3 (CC)	NA	622 (226)		**SMD= -0.61 (-0.98, -0.25)**	75% (p=0.02)	NR
	RVO		3 (CC)	NA	752 (398)		**SMD= -0.39 (-0.60, -0.19)**	31% (p=0.32)	NR
**Myocardial infarction (MI) – Supplement**									
Zhou YH (2011)	MI	Individuals with CVD or ESRD	11 (RCT)	0.5-5 mg/d for 8.3-72 months	39,923 (2,917)	Placebo	RR=1.00 (0.93, 1.07)	0% (p=0.57)	NR
Jenkins (2021)	MI	Individuals with CVD, ESRD or history of colorectal adenoma	6 (RCT)	0.5-5 mg/d for 12-64.1 months	24,210 (140)	Placebo	RR=1.24 (0.87, 1.75)	4% (p=0.23)	NR
**Revascularization – Supplement**									
Zhou YH (2011)	Revascularization	Individuals with CVD or ESRD	10 (RCT)	0.5-5 mg/d for 8.3-60 months	38,068 (NR)	Placebo	RR=1.05 (0.95, 1.16)	40% (p=0.09)	NR
**Cause-specific mortality - Supplement**									
Zhou YH (2011)	Vascular death	Individuals with CVD or ESRD	10 (RCT)	NR	27,342 (NR)	Placebo	**RR=0.89 (0.81, 0.98)**	0% (p=0.75)	NR
Jenkins (2021)	CVD mortality	Individuals with CVD, ESRD or history of colorectal adenoma	5 (RCT)	0.5-15 mg/d for 24-54 months	22,468 (201)	Placebo	RR=0.87 (0.67, 1.13)	0% (p=0.30)	NR
	MI		2 (RCT)	0.8-5 mg/d for 12-54	20,985 (13)	Placebo	RR=1.17 (0.39, 3.49)	0% (p=0.77)	NR

	mortality			months					
	Stroke mortality		2 (RCT)	0.8-5 mg/d for 12-54 months	20,985 (29)	Placebo	RR=1.85 (0.88, 3.93)	0% (p=0.11)	NR
**Total mortality – Supplement**									
Schwingshackl (2017)	All-cause mortality	Generally healthy population	2 (RCT)	0.8-5 mg/d for 37-54 months	NR (NR)	Placebo	RR=1.00 (0.60, 1.99)	37%	NR
Bazzano (2006)	Total mortality	Individuals with history of CVD or ESRD	10 (RCT)	0.5 mg/d-15g/d for 0.5-5 years	14,995 (1,821)	Placebo	RR=0.96 (0.88, 1.04)	p=0.87	No*
Zhou YH (2011)	All-cause mortality	Individuals with CVD or ESRD	11 (RCT)	0.5-5 mg/d for 8.3-72 months	44,340 (6,314)	Placebo	RR=1.00 (0.96, 1.05)	0% (p=0.91)	No*
Jenkins (2021)	All-cause mortality	Individuals with CVD, ESRD or history of colorectal adenoma	10 (RCT)	0.5-15 mg/d for 12-120 months	25,580 (895)	Placebo	RR=0.84 (0.69, 1.03)	23% (p=0.09)	NR
**VASCULAR FUNCTION SURROGATES**									
**Endothelial function – Supplement**									
Yi (2014)	All endothelial function	Individuals with CAD	6 (RCT)	5 mg/d for 4-16 weeks	1,796 (NA)	Placebo	MD= 0.00 (-0.17, 0.18)	88% (p<0.00001)	Likely*
Carotid Intima Media Thickness (CIMT)									
Qin X (2012)	IMT change	Individuals with CVD, CVD risk or CKD	4 (RCT)	0.8-15 mg/d for 3-42 months	1,459 (NA)	Placebo	**WMD= -0.04 mm (-0.06, -0.01)**	NR	NR
Flow Mediated Dilation (FMD)									
McRae (2009)	%FMD net change	Individuals with hypertension	8 (RCT)	5-10 mg/d for 2-16 weeks	372 (NA)	Placebo	MD=1.6% (1.27%, 1.96%)	NR	NR
Yi (2014)	Mean FMD	Individuals with CAD	4 (RCT)	5 mg/d for 4-16 weeks	287 (NA)	Placebo	MD=57.72 µm (50.14, 65.31)	0% (p=1.00)	NR
Liu Y (2014)	FMD	Individuals with CAD	11 (RCT)	0.4-10 mg/d for 4-16 weeks	611 (NA)	Placebo	SMD=1.65 (1.12, 2.17)	90% (p<0.00001)	0.31
Debree (2007)	%FMD net change	Individuals with CVD or CVD risk factors	10 (RCT)	0.4-10 mg/d for 4-52 weeks	712 (355)	Placebo	**Diff=1.11% (0.60, 1.62)**	NR	NR
Bokayeva (2023)	FMD	Individuals with CVD, ESRD or healthy	14 (RCT)	0.4-10 mg/d for 2-17 weeks	601 (NA)	Placebo	SMD=0.89 (0.45, 1.33)	82.9% (p<0.001)	NR
End diastolic diameter (EDD)									
Yi (2014)	Mean EDD	Individuals with CAD	4 (RCT)	5 mg/d for 4-16 weeks	237 (NA)	Placebo	MD= -0.03 (-0.20, 0.15)	0% (p=1.00)	NR
GTN (glyceryl trinitrate)-mediated dilation									
Yi (2014)	GTN diameter change	Individuals with CAD	3 (RCT)	5 mg/d for 4-16 weeks	187 (NA)	Placebo	MD=1.74 µm (-17.13, 20.61)	0% (p=0.87)	NR
***Hyperemic* flow**									
Yi (2014)	Baseline blood flow	Individuals with CAD	3 (RCT)	5 mg/d for 4-16 weeks	187 (NA)	Placebo	MD=1.02 ml/min (-4.81, 6.84)	0% (p=0.89)	NR
	Peak hyperemic flow		3 (RCT)	5 mg/d for 4-16 weeks	187 (NA)	Placebo	MD= -2.25 ml/min (-23.31, 18.82)	0% (p=0.83)	NR

**Arterial stiffness - Supplement**									
Saz-Lara (2022)	PWV	Individuals in UK with or without T2DM	2 (RCT)	5 mg/d for 4 weeks	50 (NR)	Placebo	MD= -0.14 m/s (-0.69, 0.42)	NR	NR
Bokayeva (2023)	Central PWV	Individuals with CVD, CVD risk factor or ESRD	4 (RCT)	5-15 mg/d for 4-188 weeks	421 (NA)	Placebo	SMD= -0.07 (-0.26, 0.13)	0% (p=0.90)	NR
	Peripheral PWV	Individuals with T2DM or ESRD	2 (RCT)	5-15 mg/d for 4-206 weeks	553 (NA)	Placebo	SMD= -0.09 (-0.26, 0.08)	15.2% (p=0.28)	NR
**METABOLIC RISK FACTORS**									
**Blood pressure (BP) - Supplement**									
Systolic blood pressure (SBP)									
McRae (2009)	SBP change	Individuals with hypertension	8 (RCT)	5-10 mg/d for 2-16 weeks	293 (NA)	Placebo	MD= -2.03 mmHg (-3.63, -0.43)	NR	NR
Yi (2014)	SBP	Individuals with CAD	4 (RCT)	5 mg/d for 4-16 weeks	237 (NA)	Placebo	MD= -1.07 mmHg (-5.17, 3.03)	0% (p=0.96)	NR
Tabrizi (2018)	SBP	Individuals with metabolic conditions or CVD	6 (RCT)	2.5-5 mg/d for 4-12 weeks	262 (NA)	Placebo	SMD= -0.87 (-1.83, 0.09)	92% (p<0.0001)	NR
Asbaghi (2023)	SBP	Individuals with CVD, T2DM, PCOS, HIV, postmenopausal, or alcohol/cigarette use	26 (RCT)	NR	41,633 (20,896)	Placebo	**WMD= -1.11 mmHg (-1.93, -0.28)**	65%	NR
		- baseline SBP <120 mmHg	3 (RCT)	NR	NR (NR)	Placebo	WMD= -1.03 mmHg (-3.05, 0.98)	NR	NR
		- baseline SBP ≥120 mmHg	23 (RCT)	NR	NR (NR)	Placebo	**WMD= -1.16 mmHg (-2.05, -0.27)**	NR	NR
		- individuals with CVD	14 (RCT)	NR	NR (NR)	Placebo	**WMD= -1.08 mmHg (-2.10, -0.06)**	NR	NR
		- individuals without CVD	12 (RCT)	NR	NR (NR)	Placebo	WMD= -0.97 mmHg (-2.20, 0.25)	NR	NR
		- male	4 (RCT)	NR	NR (NR)	Placebo	**WMD= -3.10 mmHg (-6.09, -0.11)**	NR	NR
		- female	5 (RCT)	NR	NR (NR)	Placebo	**WMD= -2.81 mmHg (-5.45, -0.16)**	NR	NR
		- BMI 18.5-24.9	4 (RCT)	NR	NR (NR)	Placebo	WMD= -0.53 mmHg (-1.77, 0.69)	NR	NR
		- BMI 25-29.9	15 (RCT)	NR	NR (NR)	Placebo	**WMD= -2.43 mmHg (-4.22, -0.63)**	NR	NR
Wang WW (2017)	SBP	Chinese individuals with hypertension and hyperhomocysteinemia without history of stroke or MI	49 (RCT)	0.4-10 mg/d for 8 weeks-18 months	5,707 (NA)	Antihypertensives only	**WMD= -7.85 mmHg (-9.43, -6.27)**	95% (p<0.00001)	> 0.05
Diastolic blood pressure (DBP)									
McRae (2009)	DBP change	Individuals with hypertension	8 (RCT)	5-10 mg/d for 2-16 weeks	293 (NA)	Placebo	MD= -0.01 mmHg (-1.12, 1.13)	NR	NR
Yi (2014)	DBP	Individuals with CAD	4 (RCT)	5 mg/d for 4-16 weeks	237 (NA)	Placebo	MD=0.08 mmHg (-2.10, 2.27)	0% (p=0.68)	NR
Tabrizi	DBP	Individuals with metabolic	6 (RCT)	2.5-5 mg/d for 4-12	262 (NA)	Placebo	SMD= -0.59 (-1.55,	92% (p<0.0001)	NR

(2018)		conditions or CVD		weeks			0.37)		
Asbaghi (2023)	DBP	Individuals with CVD, metabolic conditions, PCOS, HIV, or menopause	20 (RCT)	NR	41,589 (20,874)	Placebo	**WMD= -0.24 mmHg (-0.37, -0.11)**	47.9%	NR
		- baseline DBP < 80 mmHg	15 (RCT)	NR	NR (NR)	Placebo	WMD= -0.20 mmHg (-0.96, 0.54)	NR (NR)	NR
		- baseline DBP ≥ 80 mmHg	9 (RCT)	NR	NR (NR)	Placebo	WMD= -0.38 mmHg (-0.79, 0.01)	NR (NR)	NR
		- individuals with CVD	14 (RCT)	NR	NR (NR)	Placebo	**WMD= -0.23 mmHg (-0.36, -0.09)**	NR (NR)	NR
		- individuals without CVD	10 (RCT)	NR	NR (NR)	Placebo	WMD= -0.52 mmHg (-1.42, 0.37)	NR (NR)	NR
		- male	2 (RCT)	NR	NR (NR)	Placebo	**WMD= -4.19 mmHg (-7.51, -0.86)**	NR (NR)	NR
		- female	5 (RCT)	NR	NR (NR)	Placebo	WMD= -1.70 mmHg (-3.62, 0.21)	NR (NR)	NR
		- BMI 18.5-24.9	4 (RCT)	NR	NR (NR)	Placebo	**WMD= -0.30 mmHg (-0.47, -0.13)**	NR (NR)	NR
		- BMI 25-29.9	15 (RCT)	NR	NR (NR)	Placebo	WMD= -0.15 mmHg (-0.38, 0.06)	NR (NR)	NR
Wang WW (2017)	DBP	Chinese individuals with diagnosis of hypertension and hyperhomocysteinemia without history of stroke, MI	49 (RCT)	0.4-10 mg/d for 8 weeks-18 months	5,707 (NA)	Antihypertensives only	**WMD= -6.77 mmHg (-8.55, -5.00)**	97% (p<0.00001)	< 0.05
**Heart rate - Supplement**									
Yi (2014)	Heart rate	Individuals with CAD	4 (RCT)	5 mg/d for 4-16 weeks	237 (NA)	Placebo	MD= -0.39 beats/min (-2.89, 2.11)	0% (p=0.74)	NR
**Blood glucose – Supplement**									
Hemoglobin A1c (HbA1c)									
Sudchada (2012)	HbA1c	Iranian individuals with T2DM	3 (RCT)	5 mg/d for 4-8 weeks	142 (NA)	Placebo	WMD= -0.37 μmol/L (-1.10, 0.35)	83.8% (p=0.002)	NR
Zhao (2018)	HbA1c	Individuals with CVD, metabolic conditions, PCOS or cervical intraepithelial neoplasia	4 (RCT)	0.15-5 mg/d for 4 weeks - 6 months	313 (NA)	Placebo	MD= -0.17% (-0.49, 0.16)	77.8% (p=0.004)	
Akbari (2018)	HbA1c	Individuals with CVD, T2DM, T1DM, PCOS or metabolic conditions	7 (RCT)	1-10 mg/d for 4-12 weeks	309 (158)	Placebo	SMD= -0.29 (-0.61, 0.03)	40.6% (p=0.14)	No*
Lind (2019)	HbA1c	Individuals with metabolic syndrome, CVD, or cervical intraepithelial neoplasia	5 (RCT)	0.25-5 mg/d for 4-26 weeks	211 (NA)	Placebo	MD= -0.13% (-0.47, 0.20)	42% (p=0.14)	NR
Asbaghi (2021)	HbA1c	Individuals with T2DM, CVD, PCOS, endometrial hyperplasia or menopause	4 (RCT)	0.25-5 mg/d for 4-12 weeks	170 (NA)	Placebo	WMD= -0.27% (-0.73, 0.18)	74.9% (p=0.007)	NR
Fasting glucose									

Zhao (2018)	Fasting glucose	Individuals with CVD, metabolic conditions, PCOS or cervical intraepithelial neoplasia	15 (RCT)	0.15-10 mg/d for 2 weeks – 54 months	16,768 (NA)	Placebo	MD= -0.15 mmol/L (-0.29, -0.01)	53.3% (p=0.008)	
Akbari (2018)	Fasting glucose	Individuals with CVD, T2DM, T1DM, PCOS or metabolic conditions	10 (RCT)	1-10 mg/d for 4-12 weeks	511 (254)	Placebo	SMD= -0.30 (-0.63, 0.02)	69.1% (p=0.001)	No*
Lind (2019)	Fasting glucose	Individuals with CVD, T2DM, PCOS, metabolic syndrome, cervical intraepithelial neoplasia or menopause	23 (RCT)	0.4-15 mg/d for 2-26 weeks	17,242 (NA)	Placebo	MD= -0.00 mmol/L (-0.04, 0.04)	0% (p=0.89)	NR
Asbaghi (2021)	Fasting glucose	Individuals with T2DM, CVD, PCOS, endometrial hyperplasia or menopause	27 (RCT)	0.25-10 mg/d for 2-234 weeks	34,593 (NA)	Placebo	**WMD= -2.17 mg/dL (-3.69, -0.65)**	81.5% (p<0.001)	0.03
		Baseline <100 mg/dL	16 (RCT)	NR	12,547 (NA)	Placebo	**WMD= -2.14 mg/dL (-4.36, -0.06)**	85.7% (p<0.001)	NR
		Baseline ≥100 mg/dL	11 (RCT)	NR	22,067 (NA)	Placebo	**WMD= -4.06 mg/dL (-7.83, -0.29)**	71.9% (p<0.001)	NR
		Follow-up <12 weeks	17 (RCT)	NR	1,126 (NA)	Placebo	**WMD= -5.32 mg/dL (-9.11, -1.53)**	86.5% (p<0.001)	NR
		Follow-up ≥ 12 weeks	10 (RCT)	NR	33,488 (NA)	Placebo	WMD= -0.79 mg/dL (-1.81, 0.22)	48.6% (p=0.04)	NR
		FA dose < 5 mg/d	10 (RCT)	NR	33,765 (NA)	Placebo	WMD= -1.40 mg/dL (-3.23, 0.43)	84.9% (p<0.001)	NR
		FA dose ≥ 5 mg/d	17 (RCT)	NR	849 (NA)	Placebo	**WMD= -3.58 mg/dL (-6.62, -0.54)**	78.3% (p<0.001)	NR
		Individuals without T2DM	20 (RCT)	NR	21,335 (NA)	Placebo	**WMD= -2.34 mg/dL (-4.46, -0.22)**	83.7% (p<0.001)	NR
		Individuals with T2DM	7 (RCT)	NR	13,279 (NA)	Placebo	WMD= -4.87 mg/dL (-10.15, 0.39)	73.6% (p=0.001)	NR
		Female	9 (RCT)	NR	455 (NA)	Placebo	**WMD= -9.53 mg/dL (-14.71, -4.35)**	90.8% (p<0.001)	NR
		Male	2 (RCT)	NR	116 (NA)	Placebo	**WMD= -18.81 mg/dL (-26.87, -10.74)**	0% (p=0.73)	
**Insulin - Supplement**									
Zhao (2018)	HOMA-IR	Individuals with CVD, metabolic conditions, PCOS or cervical intraepithelial neoplasia	9 (RCT)	0.15-5 mg/d for 4 weeks – 6 months	435 (NA)	Placebo	MD= -0.83 units (-1.31, -0.34)	80.9% (p<0.0001)	
Akbari (2018)	HOMA-IR	Individuals with CVD, T2DM, T1DM, PCOS or metabolic conditions	9 (RCT)	1-10 mg/d for 4-12 weeks	499 (249)	Placebo	SMD= -1.07 (-1.80, -0.33)	92.5% (p<0.0001)	No*
Lind (2019)	HOMA-IR	Individuals with CV, T2DM, PCOS, metabolic syndrome, cervical intraepithelial neoplasia or menopause	9 (RCT)	1-15 mg/d for 3-26 weeks	431 (NA)	Placebo	MD= -0.49 units (-0.74, -0.24)	0% (p=0.69)	NR
Asbaghi	HOMA-IR	Individuals with T2DM, CVD,	12 (RCT)	0.3-15 mg/d for 3-25	644 (NA)	Placebo	**WMD= -0.40 (-0.70, -**	80.9% (p<0.001)	0.24

(2021)		PCOS, endometrial hyperplasia or menopause		weeks			**0.09)**		
		Follow-up < 12 weeks	5 (RCT)	NR	227 (NA)	Placebo	**WMD= -0.62 (-0.64, -0.59)**	0% (p=0.65)	NR
		Follow-up ≥ 12 week	7 (RCT)	NR	390 (NA)	Placebo	WMD= -0.31 (-0.83, 0.19)	83.7% (p<0.001)	NR
		FA dose < 5 mg/d	4 (RCT)	NR	179 (NA)	Placebo	WMD=0.02 (-0.68, 0.73)	84% (p<0.001)	NR
		FA dose ≥ 5 mg/d	8 (RCT)	NR	438 (NA)	Placebo	**WMD= -0.62 (-0.64, -0.60)**	0% (p=0.62)	NR
		Individuals without T2DM	10 (RCT)	NR	501 (NA)	Placebo	**WMD= -0.43 (-0.77, -0.08)**	83.9% (p<0.001)	NR
		Individuals with T2DM	2 (RCT)	NR	116 (NA)	Placebo	WMD=-0.26 (-0.79, 0.27)	0% (p=0.86)	NR
		Female	8 (RCT)	NR	381 (NA)	Placebo	WMD= -0.38 (-0.82, 0.06)	85.8% (p<0.001)	NR
		Male	2 (RCT)	NR	120 (NA)	Placebo	WMD= -0.26 (-0.79, 0.27)	0% (p-0.86)	NR
Zhao (2018)	Insulin	Individuals with CVD, metabolic conditions, PCOS or cervical intraepithelial neoplasia	8 (RCT)	1-15 mg/d for 3 weeks – 12 months	380 (NA)	Placebo	MD= -1.94 µl/mL (-3.28, -0.61)	66.1% (p=0.004)	
Akbari (2018)	Insulin	Individuals with CVD, T2DM, T1DM, PCOS or metabolic conditions	8 (RCT)	1-10 mg/d for 4-12 weeks	453 (226)	Placebo	**SMD= -1.28 (-1.99, -0.56)**	91.5% (p<0.0001)	No*
Lind (2019)	Fasting insulin	Individuals with CV, T2DM, PCOS, metabolic syndrome, cervical intraepithelial neoplasia or menopause	9 (RCT)	1-15 mg/d for 3-26 weeks	431 (NA)	Placebo	MD= -12.19 pmol/L (-20.49, -3.88)	44% (p=0.07)	NR
Asbaghi (2021)	Fasting insulin	Individuals with T2DM, CVD, PCOS, endometrial hyperplasia or menopause	12 (RCT)	0.4-15 mg/d for 3-25 weeks	633 (NA)	Placebo	**WMD= -1.63 µU/mL (-2.53, -0.73)**	74.9% (p=0.007)	0.96
		Follow-up < 12 weeks	6 (RCT)	NR	247 (NA)	Placebo	WMD= -1.28 µU/mL (-2.73, 0.16)	76% (p=0.001)	NR
		Follow-up ≥ 12 week	6 (RCT)	NR	359 (NA)	Placebo	**WMD= -2.03 µU/mL (-3.31, -0.75)**	55.8% (p=0.05)	NR
		FA dose < 5 mg/d	3 (RCT)	NR	148 (NA)	Placebo	**WMD= -0.99 µU/mL (-1.94, -0.04)**	0.9% (p=0.37)	NR
		FA dose ≥ 5 mg/d	9 (RCT)	NR	458 (NA)	Placebo	**WMD= -1.86 µU/mL (-3.00, -0.71)**	70.5% (p=0.001)	NR
		Individuals without T2DM	10 (RCT)	NR	490 (NA)	Placebo	**WMD= -1.96 µU/mL (-2.92, -1.00)**	65.3% (p=0.002)	NR
		Individuals with T2DM	2 (RCT)	NR	116 (NA)	Placebo	WMD=0.02 µU/mL (-1.45, 1.51)	0% (p=0.60)	NR
		Female	8 (RCT)	NR	370 (NA)	Placebo	**WMD= -2.01 µU/mL (-3.14, -0.88)**	69% (p=0.002)	NR

		Male	2 (RCT)	NR	116 (NA)	Placebo	WMD=0.02 µU/mL (-1.45, 1.51)	0% (p=0.60)	NR
**Cholesterol - Supplement**									
Tabrizi (2018)	Triglycerides	Individuals with metabolic conditions or CVD	11 (RCT)	2.5-10 mg/d for 2-12 weeks	542 (NA)	Placebo	SMD=0.10 (-0.42, 0.63)	88% (p<0.0001)	0.10
	Total cholesterol		10 (RCT)	2.5-10 mg/d for 2-12 weeks	492 (NA)	Placebo	SMD=0.06 (-0.31, 0.43)	74.9% (p<0.001)	0.08
	LDL-cholesterol		9 (RCT)	2.5-10 mg/d for 2-12 weeks	432 (NA)	Placebo	SMD= -0.14 (-0.55, 0.28)	77.1% (p<0.001)	0.37
	HDL-cholesterol		10 (RCT)	2.5-10 mg/d for 2-12 weeks	492 (NA)	Placebo	SMD=0.04 (-0.36, 0.44)	78.7% (p<0.0001)	0.16
	VLDL-cholesterol		3 (RCT)	2.5-5 mg/d for 8-12 weeks	145 (NA)	Placebo	SMD=0.08 (-0.24, 0.41)	0% (p<0.65)	0.28
**Body weight – Supplement**									
Jafari (2023)	Body weight	Individuals with T2DM, PCOS or menopause	7 (RCT)	1.5-15 mg/d for 3-24 weeks	378 (NA)	Placebo	WMD= -0.16 kg (-0.47, 0.16)	40.4% (p=0.12)	0.38
**Body Mass Index (BMI) – Supplement**									
Jafari (2023)	BMI	Individuals with T2DM, PCOS or menopause	9 (RCT)	1.5-7.5 mg/d for 3-12 weeks	436 (NA)	Placebo	WMD= -0.23 kg/cm^2 (-0.49, 0.03)	90.1% (p<0.001)	0.11
		Homocysteine ≥ 15 µmol/L	NR	NR	NR (NR)	Placebo	WMD= -0.17 kg/cm^2 (-0.33, 0.01)	NR	NR
		Women with PCOS	NR	NR	NR (NR)	Placebo	**WMD= -0.30 kg/cm^2 (-0.54, -0.06)**	NR	NR
**Polycystic Ovary Syndrome (PCOS) – Dietary folate**									
Kazemi (2022)	PCOS	Women aged 18-50 years	5 CS, 4 CC	NA	9,562 (1,161)	Healthy control	**MD= -20.80 µg/d (-42.65, 1.05)**	31.5%	<.001
**All outcomes - Dose-response analyses**									
Jayedi (2019)	CHD	Individuals without history of CVD	7 (PC)	NA	212,284 (4,022)	NA	**RR=0.79 (0.69, 0.89) per 250 µg/d increase in dietary or total folate intake**	67%	
Wang ZM (2012)	CHD	General population	7 (PC)	NA	223,691 (2,682)	NA	**RR=0.88 (0.82, 0.94) per 200 µg/d increase in dietary folate intake**	27.4% (p=0.22)	
Wang ZM (2012)	CHD	General population	8 (PC)	NA	14,533 (1,936)	NA	**RR=0.92 (0.84, 1.00) per 5 mmol/L increase in plasma folate concentration**	51.7% (p=0.03)	
Chen (2020)	Stroke	Individuals with CVD	8 (PC)	NA	253,511 (7,429)	NA	**RR=0.94 (0.90, 0.98) per 100 µg/d increase in dietary folate intake**	46.8%	

ACKD: advanced chronic kidney disease; AMI: acute myocardial infarction; BMI: body mass index; BP: blood pressure; CAD: coronary artery disease; CC: case-control study; CHD: coronary heart disease; CIMT: carotid intima media thickness; CS: cross-sectional study; CVCE: cardiovascular and cerebrovascular event; CVD: cardiovascular disease; CVT: cerebral venous thrombosis; DBP: diastolic blood pressure; DVT: deep vein thrombosis; EDD: endothelium-dependent dilation; ESRD: end-stage renal disease; FMD: flow-mediated dilation; GTN: glyceryl trinitrate; MI: myocardial

infarction; NA: not applicable (continuous variable); NCC: nested case-control study; NR: not reported; PCOS: polycystic ovarian syndrome; PTE: pulmonary thromboembolism; PWV: pulse wave velocity; RVO: retinal vein occlusion; SBP: systolic blood pressure; T2D: type 2 diabetes; VT: venous thrombosis. **Bolded**: statistically significant. * the authors reported no publication bias based on visual inspection

Supplementary Table 3. Risk of bias assessment of the syntheses included in this review

Synthesis	Domain 1: Study eligibility criteria	Domain 2: Identification and selection of studies	Domain 3: Data collection and study appraisal	Domain 4: Synthesis and findings	Risk of Bias
Akbari (2018)	⊕	⊕	⊖	⊕	⊕
Asbaghi O (2021)	⊕	⊕	⊕	⊕	⊕
Asbaghi (2023)	⊕	⊕	⊕	⊖	⊕
Bartlett HE (2008)	⊗	⊖	⊗	⊗	⊗
Bazzano (2006)	⊕	⊗	⊗	⊕	⊗
Bokayeva K (2023)	⊕	⊕	⊕	⊕	⊕
Chen L (2020)	⊕	⊕	⊕	⊕	⊕
Colapinto CK (2016)	⊖	⊕	⊕	⊕	⊖
deBree A (2007)	⊕	⊗	⊕	⊕	⊗
Dong H (2015)	⊕	⊕	⊕	⊕	⊕
Eikelboom JW (1999)	⊗	⊗	⊗	⊗	⊗
Foroughi M (2013)	⊗	⊗	⊗	⊗	⊗
Heinz J (2009)	⊕	⊕	⊗	⊗	⊗
Hsu CY (2018)	⊕	⊕	⊕	⊕	⊕
Huo Y (2012)	⊕	⊗	⊕	⊕	⊕
Jafari A (2023)	⊕	⊕	⊕	⊕	⊕
Jayedi A (2019)	⊕	⊖	⊕	⊗	⊗
Jenkins DIA (2021)	⊗	⊕	⊕	⊖	⊗
Kazemi M (2022)	⊕	⊕	⊕	⊕	⊕
Khandanpour N (2009)	⊕	⊕	⊗	⊖	⊗
Lee M (2010)	⊕	⊕	⊗	⊕	⊕
Li Y (2016)	⊕	⊕	⊕	⊕	⊕
Lind MV (2019)	⊕	⊕	⊕	⊕	⊕

Liu Y (2014)	⊕	⊕	⊕	⊕	⊕
McRae MP (2009)	⊕	⊗	⊗	⊗	⊗
Mente A (2009)	⊕	⊗	⊗	⊗	⊗
Myung SK (2013)	⊕	⊕	⊖	⊕	⊕
Pan Y (2012)	⊕	⊗	⊗	⊗	⊗
Pase M (2011)	⊕	⊕	⊕	⊕	⊕
Qin X (2011)	⊕	⊗	⊖	⊕	⊖
Qin X (2012)	⊕	⊗	⊕	⊕	⊕
Qin X (2013)	⊕	⊗	⊕	⊖	⊖
Saz-Lara A (2022)	⊕	⊕	⊕	⊕	⊕
Schwingshackl L (2017)	⊕	⊕	⊕	⊕	⊕
Sudchada P (2012)	⊕	⊕	⊕	⊕	⊕
Tabrizi R (2018)	⊕	⊗	⊕	⊕	⊗
van Hattum ES 2007	⊕	⊗	⊕	⊗	⊗
Wang WW (2017)	⊕	⊗	⊕	⊕	⊖
Wang X (2007)	⊕	⊕	⊗	⊗	⊗
Wang Y (2019)	⊕	⊖	⊕	⊕	⊖
Wang ZM (2012)	⊕	⊖	⊕	⊕	⊖
Yi X (2014)	⊕	⊗	⊕	⊕	⊖
Zeng R (2015)	⊕	⊕	⊗	⊕	⊗
Zhang C (2013)	⊕	⊕	⊕	⊕	⊕
Zhang C (2014)	⊕	⊕	⊕	⊕	⊕
Zhao JV (2018)	⊕	⊕	⊕	⊕	⊕
Zhao M (2017)	⊕	⊗	⊕	⊕	⊕
Zhou K (2012)	⊕	⊕	⊕	⊕	⊕
Zhou YH (2011)	⊕	⊖	⊕	⊕	⊕
High risk of bias	4	16	12	10	16
Low risk of bias	44	28	34	35	26
Unclear risk of bias	1	5	3	4	7
